# Biochemical characterization of the *Nocardia lactamdurans* ACV synthetase

**DOI:** 10.1371/journal.pone.0231290

**Published:** 2020-04-10

**Authors:** Riccardo Iacovelli, Reto D. Zwahlen, Roel A. L. Bovenberg, Arnold J. M. Driessen

**Affiliations:** 1 Department of Molecular Microbiology, Groningen Biomolecular Sciences and Biotechnology Institute, University of Groningen, Groningen, The Netherlands; 2 Synthetic Biology and Cell Engineering, Groningen Biomolecular Sciences and Biotechnology Institute, University of Groningen, Groningen, The Netherlands; 3 DSM Biotechnology Centre, Delft, The Netherlands; Weizmann Institute of Science, ISRAEL

## Abstract

The L-δ-(α-aminoadipoyl)-L-cysteinyl-D-valine synthetase (ACVS) is a nonribosomal peptide synthetase (NRPS) that fulfills a crucial role in the synthesis of β-lactams. Although some of the enzymological aspects of this enzyme have been elucidated, its large size, at over 400 kDa, has hampered heterologous expression and stable purification attempts. Here we have successfully overexpressed the *Nocardia lactamdurans* ACVS in *E*. *coli* HM0079. The protein was purified to homogeneity and characterized for tripeptide formation with a focus on the substrate specificity of the three modules. The first L-α-aminoadipic acid-activating module is highly specific, whereas the modules for L-cysteine and L-valine are more promiscuous. Engineering of the first module of ACVS confirmed the strict specificity observed towards its substrate, which can be understood in terms of the non-canonical peptide bond position.

## Introduction

Nonribosomal peptides (NRP) represent a very versatile group of low to medium molecular weight compounds that exhibit various biological activities. These peptides are exclusively produced by nonribosomal peptide synthetases (NRPS) and do not only contain proteinogenic amino acids, but may also contain a wide variety of non-proteinogenic amino acids and hydroxy acids [1]. NRP often undergo a series of modifications *in cis*, whether through the action of the NRPS or by further tailoring enzymes.

NRP synthesis universally starts in every module with the adenylation (A) domain, serving as a highly selective gate keeper, which recruits and adenylates a specific substrate, thereby forming an acyl-adenylate conjugate. Subsequently, the substrate-conjugate is transferred to the thiolation (T) domain by means of the phosphopantetheine (ppant) arm, with the AMP being released. The ppant arm is a CoA (Coenzyme A)-derived cofactor, covalently attached to a highly conserved residue of serine of the T domain by a ppant-transferase. The activated substrates are then transported to the donor and acceptor sites of the up- or downstream condensation (C) domains, where peptide formation occurs with the upstream substrate being released from the ppant moiety, and the newly synthesized intermediate ready to be transported to the next condensation domain [2–6]. In some cases, the growing peptide can be further modified by accessory domains, such as N-methylation, substrate epimerization, and heterocyclization domains [7][8]. When the synthesis is completed, the peptide is often released via the activity of a thioesterase (Te) domain, either by macrocyclization or hydrolysis, resulting in a cyclic or a linear NRP, respectively [8][9].

Despite extensive efforts, including the solution of sub-domain, domain, di-domain and entire modular structures [5,10–14], the high conformational dynamics and flexibility that characterize NRPS enzymes [15] have rendered structural analysis a considerable challenge. Only recently the first structures of a dimodular NRPS were obtained [16], providing crucial information on the dynamics of inter-domain and inter-module interactions and, ultimately, NRP synthesis.

**Fig 1:**
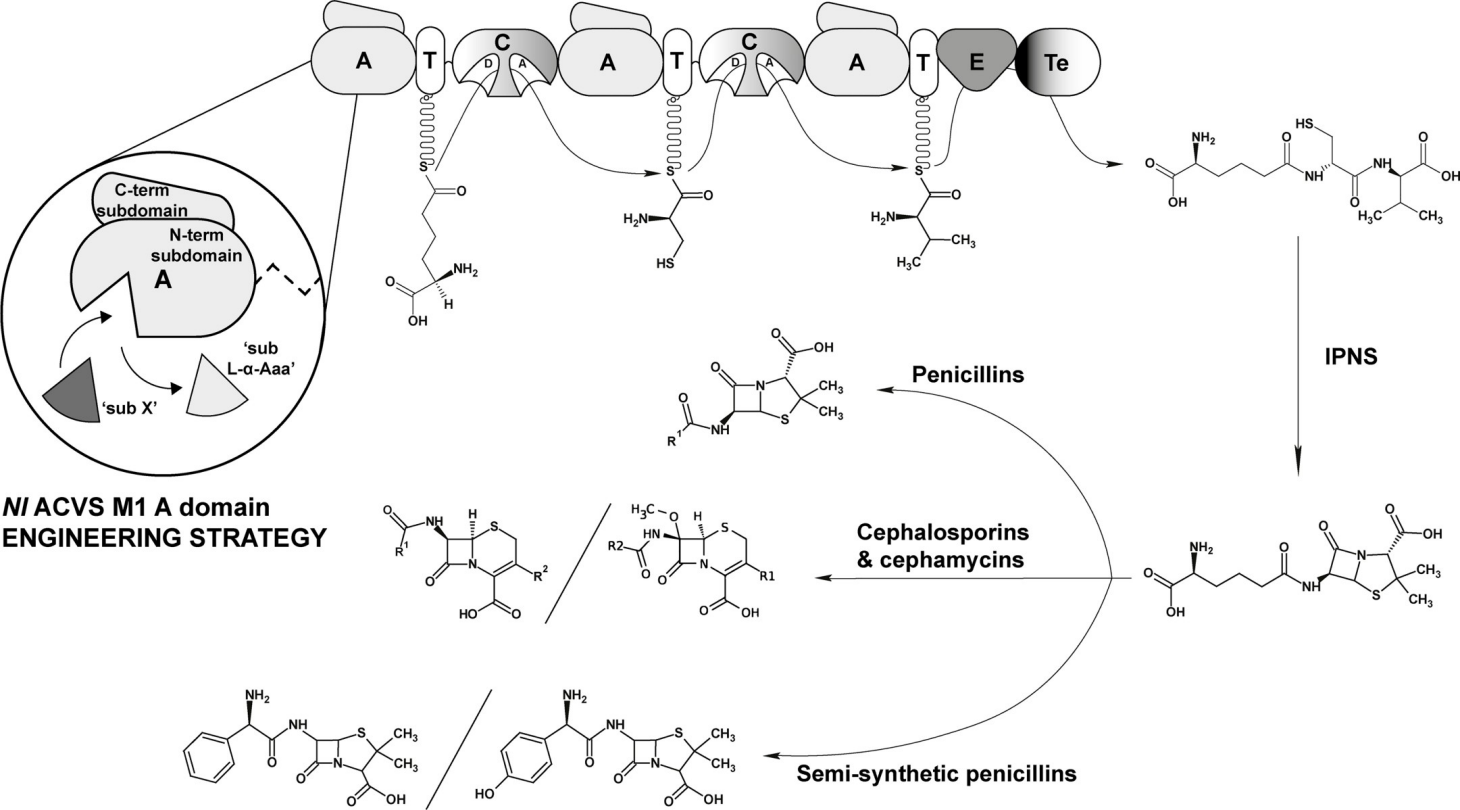
ACVS domain organization and product formation. The ACVS consists of a total of 10 domains arranged in three modules with distinct specificities for the incorporation of L-α-aminoadipic acid (L-α-Aaa), L-cysteine and L-valine into the tripeptide δ-(L-α-aminoadipoyl)-L-cysteinyl-D-valine ((L,L,D)-ACV). The domain arrangement is conserved [30] and follows the order: AT-CAT-CATETe. The resulting (L,L,D)-ACV is converted into isopenicillin-N (IPN) by the isopenicillin-N synthetase (IPNS). Following different biosynthetic routes, IPN can further be converted into penicillins, cephalosporins, cephamycins and related compounds. In the circle, a schematic representation of the strategy [33] adopted to engineer the specificity of the first module of *Nl* ACVS is shown.

Due to the relative simplicity and overall significance, the β-lactam production pathway [17–22] has been a paradigm for related research fields. Three distinct enzymatic steps are involved in the production of β-lactams, with the trimodular NRPS L-δ-(α-aminoadipoyl)-L-cysteinyl-D-valine synthetase (ACVS) providing the tripeptide (L,L,D)-ACV as the precursor for β-lactam antibiotics, such as penicillins or cephalosporins (Fig 1). The three amino acids L-α-aminoadipic acid (L-α-Aaa), L-cysteine and L-valine are inserted in the final product in a co-linear fashion, thus the position of the incorporated substrate corresponds to the position of the respective module within the primary NRPS sequence [23]. Peptide formation itself is strictly determined by the selectivity of the domains of the ACVS. Furthermore, L-α-aminoadipic acid is adenylated on the δ-carboxyl group, resulting in a non-canonical peptide bond formation between L-α-Aaa and L-Cys. Lastly, L-valine is epimerized via an intrinsic epimerization (E) domain, located in the third module. The final product is released as a linear tripeptide, (L,L,D)-ACV, by a Te domain, and it is subsequently converted to isopenicillin N by the enzyme isopenicillin N synthase (IPNS), which catalyzes the formation of the β-lactam ring.

ACVS served as a model NRPS, aiding to establish the nonribosomal route of peptide production. The protein has been studied in filamentous fungi such as *Penicillium*, *Aspergillus*, *Cephalosporium* as well as bacterial *Nocardia* and *Streptomyces* species [24–30]. The partial biochemical reactions of the NRPS domains have been examined mostly by using crude cell extracts or partially purified enzyme [30]. The large size of the protein, 404–425 kDa [30], makes expression and purification challenging for biochemical characterization. Here, we focus on the *pcbAB* gene of *Nocardia lactamdurans* that encodes a 404 kDa ACVS, first enzyme of the cephamycin biosynthetic pathway in this organism [29]. This protein was previously overexpressed in *Streptomyces lividans*, purified to near homogeneity and characterized for ACV synthetase activity. The enzyme activity was measured using ^14^C‐valine in an ATP/PP_i_ exchange assay [31]. Here, we heterologously overexpressed *Nl* ACVS in *E*. *coli* HM0079 [32], a platform strain that carries the 4′-phosphopantetheine transferase gene *sfp*, crucial for the production of active holoenzymes. The protein was purified to homogeneity and characterized for tripeptide production and substrate promiscuity via HPLC-MS. This allowed for the determination of fundamental biochemical parameters and substrate specificity of the individual modules. Furthermore, we engineered the adenylation domain of the first module of ACVS, adapting a subdomain swap strategy [33][34] with the goal of generating hybrid NRPSs able to activate alternative substrates and incorporate these at the first position of the tripeptide for novel β-lactam production.

## Results

### Purification and biochemical characterization of the *N*. *lactamdurans* ACVS

**Fig 2:**
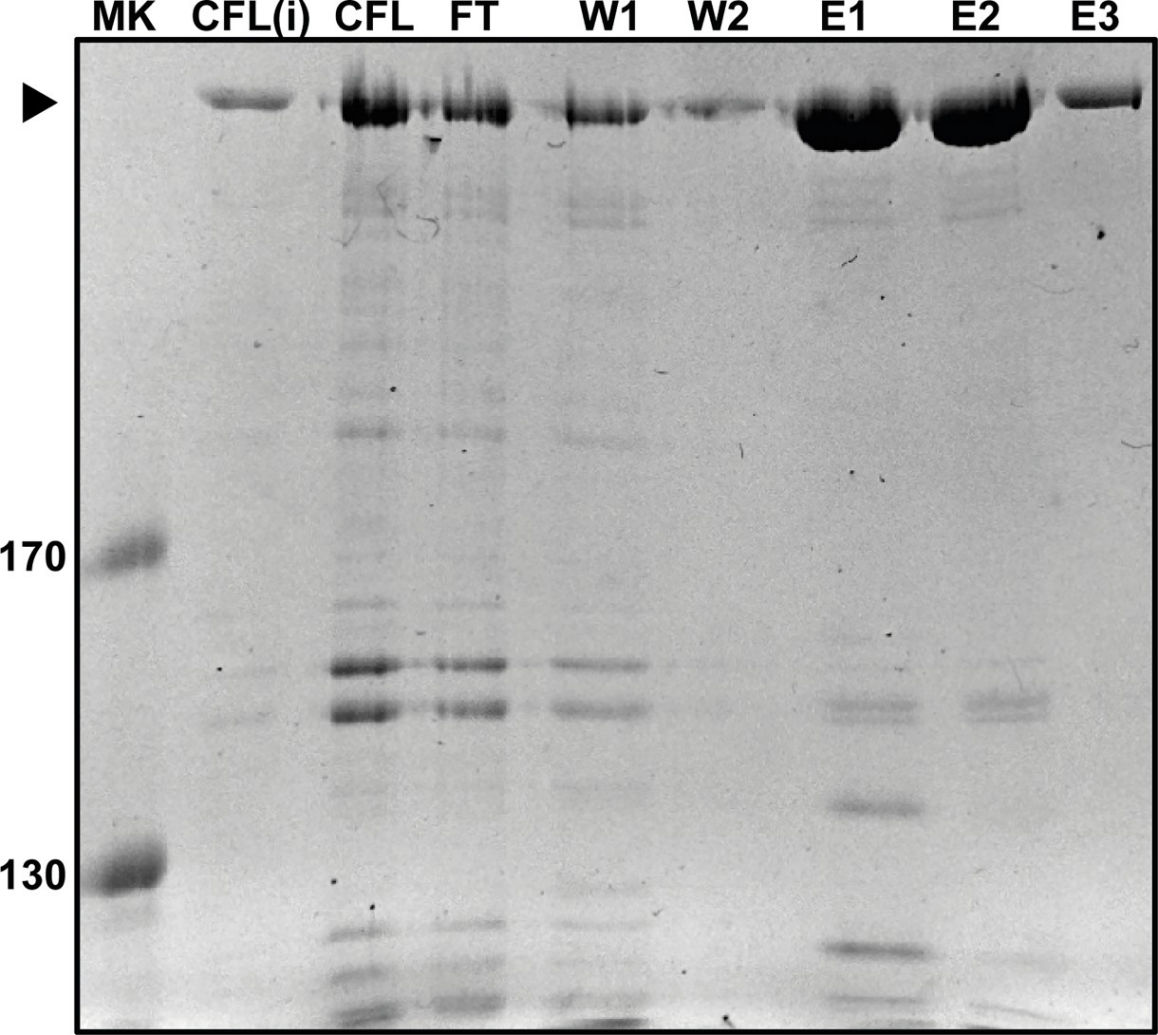
Ni^2+^ affinity chromatography purification of the *Nocardia lactamdurans* ACVS. *Nl* ACVS was isolated from *E*. *coli* HM0079 cells and harvested after overnight expression at 18 ºC. A cell-free lysate was obtained through sonication and subsequently separated into a clear supernatant (CFL) and the pellet was resuspended in 8 M Urea (CFL (i)). The clear lysate was further purified using gravity flow in combination with a His-tag affinity chromatography, using two washing steps (W1, W2) and elution with 50, 150 and 250 mM imidazole, respectively (E1, E2, E3). Marker lane shows reference proteins corresponding to 170 and 130 kDa.

**Fig 3:**
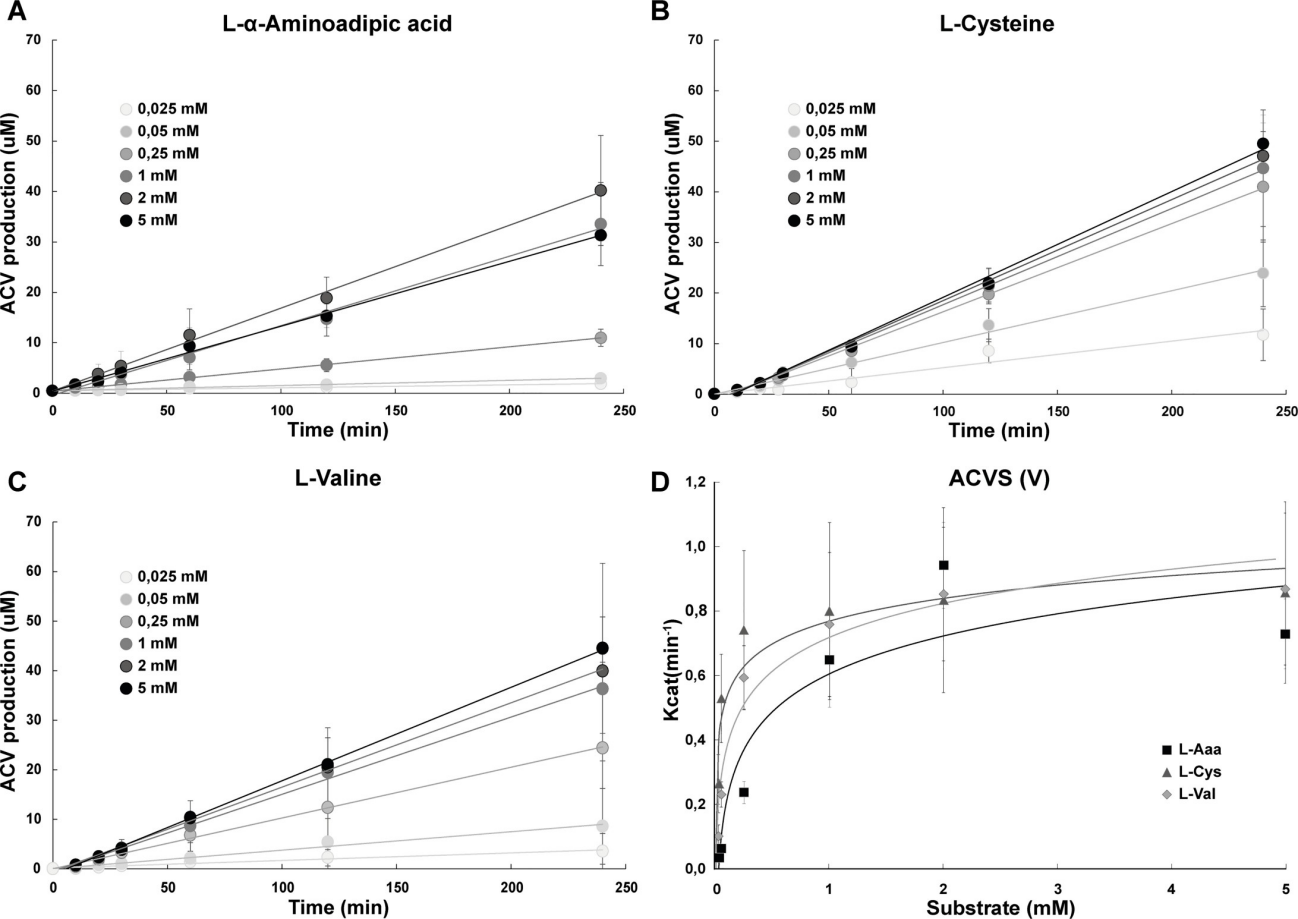
Enzymatic characterization of ACVS. Three reaction series were conducted using various concentrations of L-α-Aaa (A), L-Cys (B) and L-Val (C) and analyzed by LC/MS to quantify the amounts of the ACV tripeptide produced for Michaelis-Menten kinetics (D).

The *Nocardia lactamdurans* ACVS was overexpressed in *E*. *coli* HM0079 as a C-terminal 6xHis-tagged protein, and purified by Ni^2+^ affinity purification. The overall yield was 13.9 ± 3.4 mg pure ACVS per liter of culture (Fig 2). The purified enzyme (fraction E3) was subjected to *in vitro* product formation assays using conditions outlined in the methods section. In these assays, varying concentrations of the three substrate amino acids were used (Fig 3). Reactions were evaluated over a 4 h time course, and analyzed by HPLC/MS. Resulting ACV levels were quantified and normalized, showing a near linear product formation over the entire time course (Fig 3A–3C). Maximal ACV product levels under the given conditions reached nearly 50 μM, exceeding the enzyme concentration (0.17 μM) by almost three orders of magnitude, indicating multiple turnovers. The calculated V_max_ value for the ACVS activity was 0.78 ± 0.14 μM (ACV)*min^-1^*μM enzyme^-1^. Apparent K_M_ values were determined from the Michaelis-Menten kinetics with a >98% curve fit. Values of 640 ± 16, 40 ± 1 and 150 ± 4 μM were obtained for L-α-aminoadipic acid, L-cysteine and L-valine, respectively (Fig 3D).

### Substrate specificity of the *Nl* ACVS

**Fig 4:**
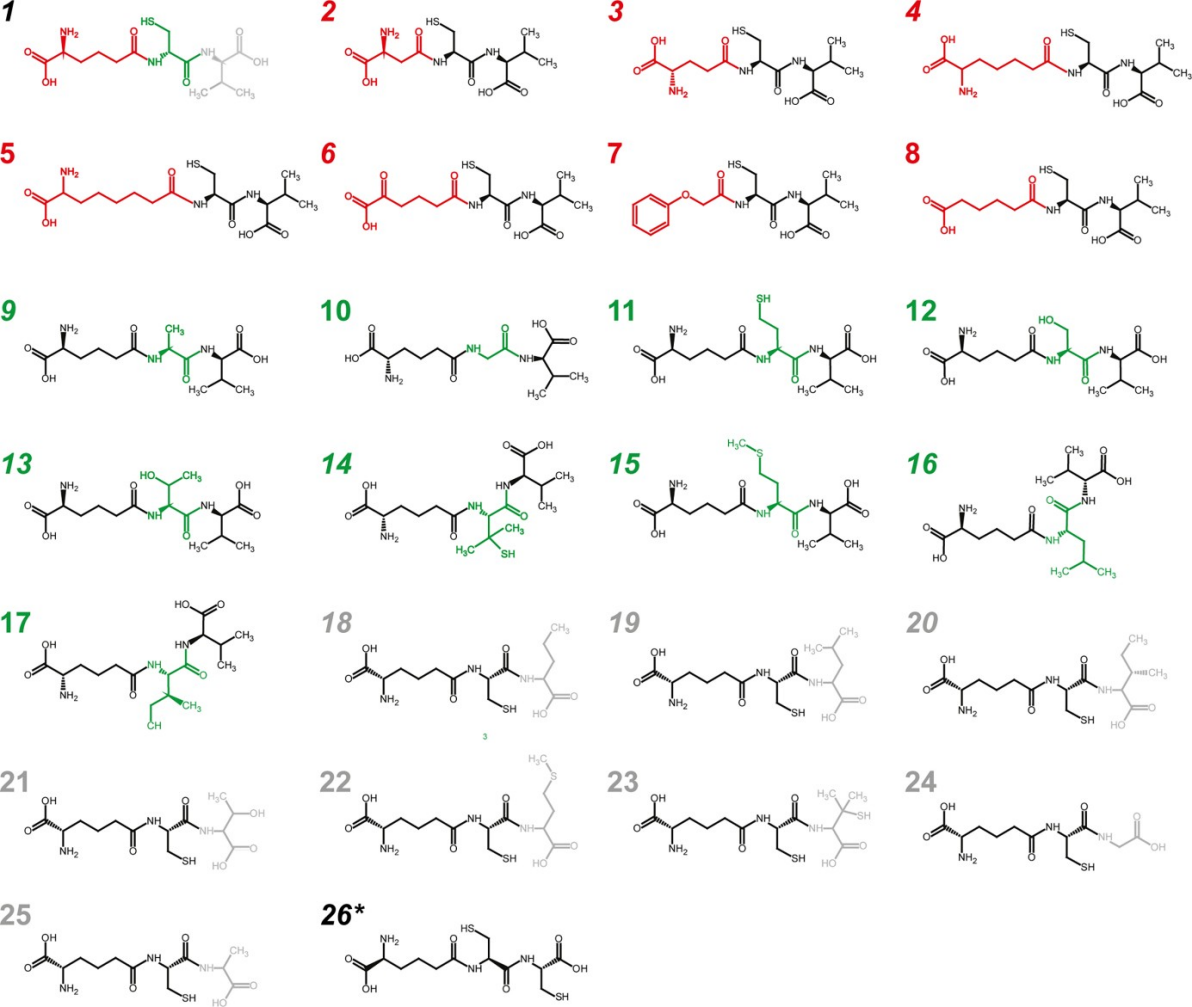
Substrate promiscuity of the *N*. *lactamdurans* ACVS: Structures of expected tripeptides. The predicted structures of the novel tripeptides and their corresponding reactions are numbered 1–26 (see Table 1). Italic numbers indicate production of the respective tripeptide. * = ACC tripeptide derived from L-α-Aaa and L-Cys only (26).

Next, we determined the enzyme substrate specificity. Therefore, three sets of reactions were arranged, varying the substrate for each of the three ACVS modules, also using structural analogues. The concentration of the variable amino acid was set at 5 mM. Product levels were determined as end points after 4h and analyzed for formation of the predicted tripeptides and related structures using HPLC/MS (Fig 4). In addition to the three native substrates, 17 analogues were tested in a total of 25 reaction setups (Table 1). Next to the ACV tripeptide, we detected 11 of the expected tripeptides (*M1*: 3; *M2*: 5; *M3*: 3) as well as the AC-Cys tripeptide in a reaction using L-α-Aaa and L-Cys only (S1 Appendix). Based on extracted ion count, production levels vary strongly for the various tripeptides, from 0.003% up to 13.8% relative to ACV production levels, assuming the same degree of ionization for the alternative tripeptides, for which no chemical standard was available (Table 1).

**Table 1:** ACVS substrate promiscuity.

	Analogue	Tripeptide	#	Mi	rel prod (±err)
**ACVS**	*-*	(L,L,D)-ACV	**1**	363,146	100 ± 10.3
**M1**	L-Aspartic acid	Asp-CV	**2**	335,115	0
	L-Glutamic acid	Glu-CV	**3**	349,131	0.02 ± 0.009
	DL-aminopimelic acid	API-CV	**4**	377,162	0.01
	DL-aminosuberic acid	ASU-CV	**5**	391,178	0
	2-oxoadipic acid	OAA-CV	**6**	362,115	0.003
	Phenoxyacetic acid	POA-CV	**7**	354,125	0
	Adipic acid	AA-CV	**8**	348,136	0
**M2**	L-Alanine	A-Ala-V	**9**	331,174	0.98 ± 0.02
	L-Glycine	A-Gly-V	**10**	317,159	0
	DL-Homocysteine	A-Hcys-V	**11**	377,162	0
	L-Serine	A-Ser-V	**12**	347,169	0
	L-Threonine	A-Thr-V	**13**	361,185	0.02 ± 0.003
	L-Penicillamine	A-Pen-V	**14**	391,178	0.05 ± 0.005
	L-Methionine	A-Met-V	**15**	391,178	0.07 ± 0.01
	L-Leucine	A-Leu-V	**16**	373,221	1.58 ± 0.21
	L-Isoleucine	A-Ile-V	**17**	373,221	0
**M3**	L-Norvaline	AC-NorVal	**18**	363,146	13.8 ± 0.59
	L-Leucine	AC-Leu	**19**	377,162	0.54 ± 0.01
	L-Isoleucine	AC-Ile	**20**	377,162	1.21 ± 0.04
	L-Threonine	AC-Thr	**21**	365,126	0
	L-Methionine	AC-Met	**22**	395,118	0
	L-Penicillamine	AC-Pen	**23**	395,118	0
	L-Glycine	AC-Gly	**24**	321,099	0
	L-Alanine	AC-Ala	**25**	335,115	0
	*L-Cysteine*	AC-Cys*	**26**	367,087	13.6 ± 1.93

Three sets of reactions were analyzed varying the amino acid on one position within the tripeptide. Alternative substrates were added to a concentration of 5 mM, replacing either **L-α-Aaa** (M1), **L-Cys** (M2) or **L-Val** (M3). Reactions (numbered 1–26) were evaluated using LC/MS and peaks of interest were assessed according to accurate monoisotopic mass (Mi). The resulting levels were set relative to the production of ACV (= 100), assuming similar ionization. Values derived from two biological and technical replicates ± standard deviation (except for DL-aminopimelic acid and 2-oxoadipic acid reactions, with only two technical replicates).

### Engineering of the first adenylation domain of *Nl* ACVS

**Fig 5:**
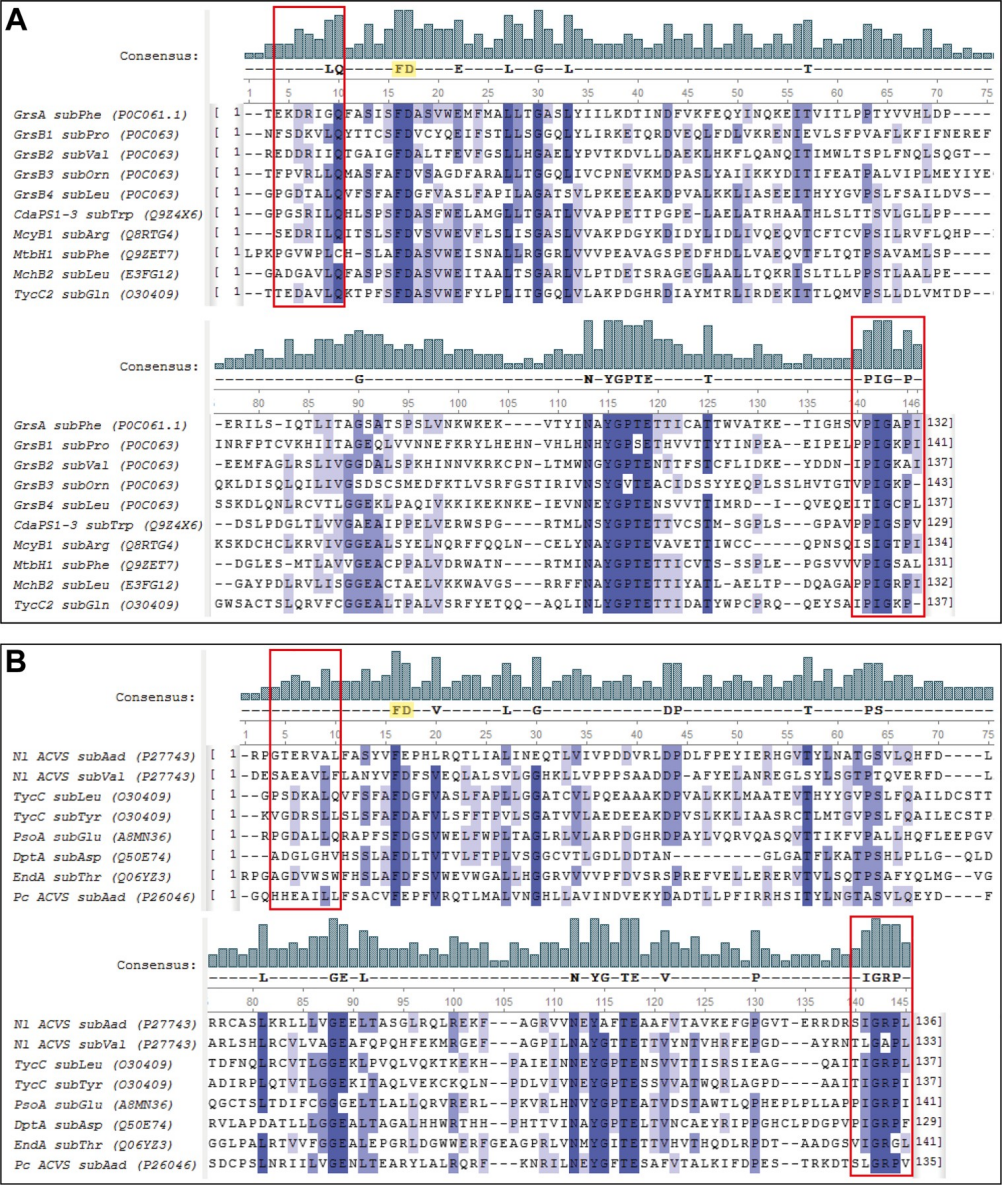
Subdomains multi-alignment and boundaries determination. (A) Multi-alignment of the subdomains used in the engineering strategy proposed by Kries and coworkers [33]. The two residues highlighted in yellow in the consensus sequence represent the first highly conserved motif with a phenylalanine and the aspartic acid which forms a hydrogen bond with the α-amino group of the amino acid substrate [35]. The motifs highlighted with the red boxes represent the conserved motifs identified as boundaries of the subdomains in the consensus sequence. (B) Multi-alignment of the subdomains selected for this work; in absence of structural information the conserved motifs at both ends were used to determine the subdomain boundaries.

Next, we constructed a set of hybrid NRPS enzymes, by replacing the L-α-aminoadipic acid-specific subdomain of the first adenylation domain of *Nl* ACVS with alternative amino acid sequences from donor NRPSs (S1 Fig). Herein, the word subdomain is used to define specific regions of the adenylation domains which meet distinct criteria, determined by Kries and coworkers [33]. Donor NRPSs were selected according to their substrate specificities: the alternative sequences were chosen to explore the possibility to engineer hybrids that would activate and incorporate amino acids with different types of side chains (L-glutamic acid, L-aspartic acid, L-threonine, L-leucine, L-tyrosine and L-valine). One of the subdomains (L-valine-specific) was selected from the third module of *Nl* ACVS itself. We further included the L-α-aminoadipic acid-specific subdomain from the first module of *Penicillium chrysogenum* ACVS, with a subdomain sequence identity of 48.5%, as a control. The complete set of subdomains used in the engineering strategy is listed in Table 2. The amino acid sequences encoding the substrate-specific subdomains were identified through multi-sequence alignment analysis, as outlined in the methods section and in Fig 5. Hybrid NRPS constructs were built using a system (S2 Fig) based on the Golden Gate assembly, a synthetic biology method that allows easy and seamless assembling of smaller DNA fragments into larger genes [34]. All the intermediate vectors utilized in the assembly strategy were successfully cloned in *E*. *coli* DH5α and sequenced individually. The Golden Gate assembly reactions for the hybrid NRPSs, which were named ECVS, DCVS, TCVS, LCVS, YCVS, VCVS and *Pc*A*Nl*CVS (according to the predicted specificity of the hybrid module), were performed *in vitro* and transformed directly into *E*. *coli* DH5α cells for storage and sequencing.

**Table 2:** Set of subdomains selected for the engineering strategy.

Subdomain ID	Parent NRPS	UniProtKB Accession No.	Organism of origin	subdomain boundaries (aa)
*Nl* ACVS subAad	ACV synthetase	P27743	*Nocardia lactamdurans*	442–577
*Nl* ACVS subVal	ACV synthetase	P27743	*Nocardia lactamdurans*	2583–2715
TycC subLeu	Tyrocidine synthase 3	O30409	*Brevibacillus parabrevis*	5839–5975
TycC subTyr	Tyrocidine synthase 3	O30409	*Brevibacillus parabrevis*	2718–2854
PsoA subGlu	PsoA	A8MN36	*Pseudomonas putida*	1702–1742
DptA subAsp	DptA	Q50E74	*Streptomyces filamentosus*	3271–3399
EndA subThr	EndA	Q06YZ3	*Streptomyces fungicidicus*	1686–1826
*Pc* ACVS subAad	ACV synthetase	P26046	*Penicillium chrysogenum*	515–649

**Fig 6:**
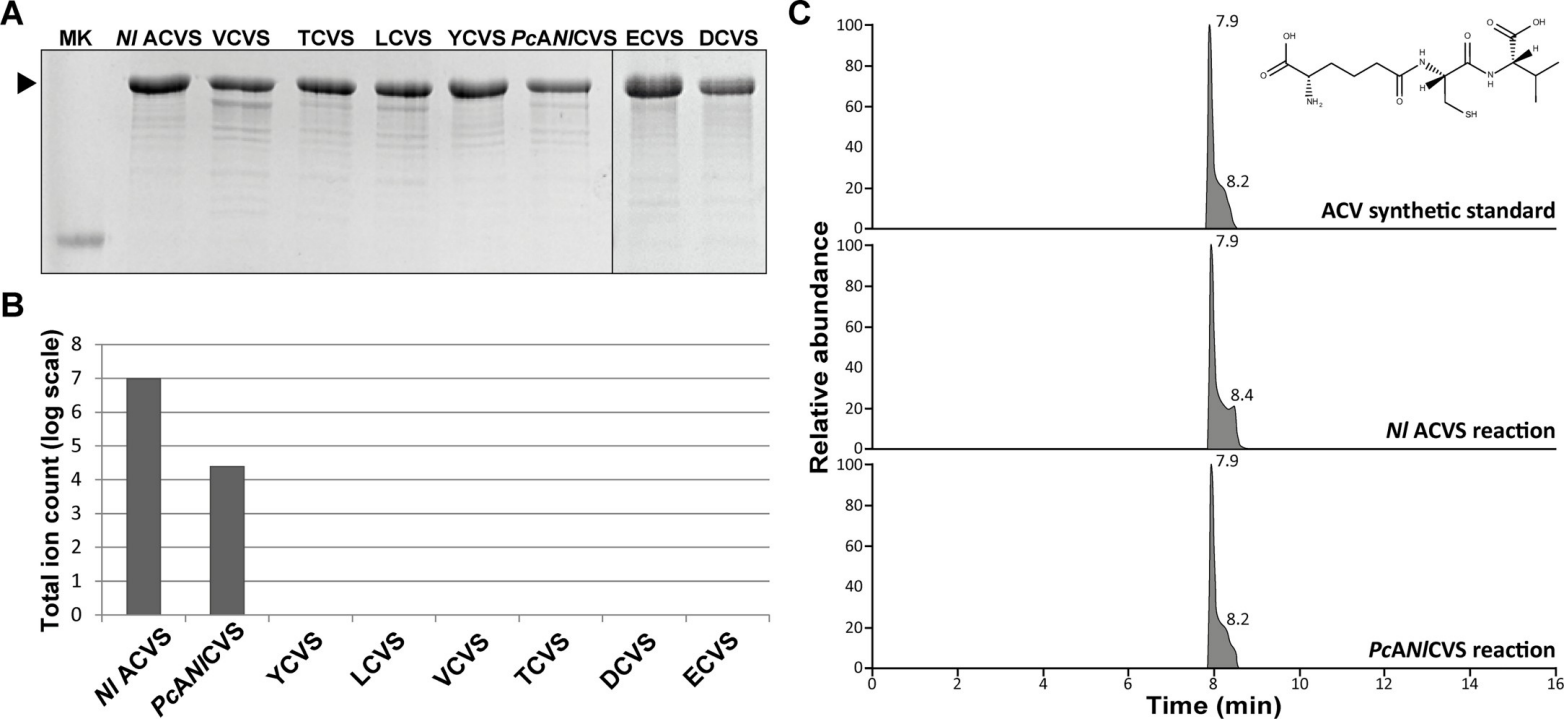
Overexpression and activity assays of the hybrid NRPSs. (A) SDS-PAGE analysis of the purified fractions of the hybrids: only fraction E2 (150 mM imidazole) is shown. Marker lane shows reference protein corresponding to 180 kDa. (B) *In vitro* tripeptide production assays with hybrid enzymes. (C) The production of the tripeptide ACV was confirmed by accurate monoisotopic mass and equal retention time compared to the wild-type enzyme product and an ACV synthetic standard.

Hybrid NRPS genes were overexpressed in *E*. *coli* HM0079 as C-terminal 6xHis-tagged proteins, and purified by Ni^2+^ affinity purification as described for the wild-type *Nl* ACVS. The overall yield was slightly lower compared to the *Nl* ACVS, ranging between 4 and 9 milligrams of pure protein per liter of culture (Fig 6A). Next, the purified hybrids were subjected to *in vitro* product formation assays including the amino acid substrates required for product formation. The full HPLC/MS chromatograms were analyzed and filtered for the m/z values specific to the predicted tripeptides (S3 Fig). Only in one case we detected the production of the expected compound. The hybrid *Pc*A*Nl*CVS produced the ACV tripeptide, though at considerably lower levels compared to the wild-type *Nl* ACVS (Fig 6, panels B and C).

## Discussion

Here we report on the characterization of the *Nocardia lactamdurans* ACV synthetase, heterologously overexpressed in *E*. *coli* HM0079 [32] and purified to homogeneity. The aforementioned *E coli* strain contains a genomic copy of *sfp*, a phosphopantheteinyl transferase, essential to activate the apo-ACVS to its active holo-form. An efficient purification process was developed to obtain highly pure enzyme. Initial enzymatic characterization was performed by following the production of the tripeptide ACV. With respect to the three ACVS modules, distinct differences in substrate affinities were noted with the initiating L-α-aminoadipic acid module showing the lowest affinity for its substrate. Previous studies on the formation of product intermediates and partial reactions of NRPS enzymes suggest that the initial amino acid thiolation reaction is a rate limiting step in the assembly of nonribosomal peptides, necessary for the subsequent domains to adopt their distinct conformations for peptide bond formation and product release [6][36]. Overall, substrate affinities levels appear to be in line with other ACVS homologues, in particular prokaryotic enzymes [37].

We furthermore determined the substrate specificity of the ACVS modules within the context of the complete enzyme, by assessing the production of predicted tripeptides (Table 1 and Fig 4). Some ACVS homologues [31,38–40] exhibit a certain degree of substrate tolerance, despite considerably tight control mechanisms that assure correct product formation. With the *N*. *lactamdurans* ACVS, replacement of L-α-aminoadipic acid by substrate analogues yielded only trace amounts of Glu-CV, previously reported [38,40], and API-CV and OAA-CV tripeptides, while none of the other substrates tested were incorporated. The structures of the three analogues activated strongly resemble that of L-α-Aaa, thus indicating a strict specificity for module 1. With the second module (L-cysteine), two alternative tripeptides A-Ala-V and A-Leu-V were generated in substantial amounts while trace amounts of three other A-X-V tripeptides were found (Table 1 and Fig 4). This suggests that this module is more promiscuous. Finally, for the third module (L-valine), two novel products AC-Leu and AC-Ile were observed at a level of 0.5–1%, while AC-NorVal was found at levels over 13% relative to ACV. Module 3 shows some tolerance towards side chain length and the distribution of methyl-groups. However, substrates with hydroxyl or thiol groups are not incorporated. In addition, in the absence of L-valine, we observed the production of a putative AC-Cys tripeptide up to a level of 13%. While these results indicate that the individual modules can accept only certain substrates, it cannot be entirely excluded that some tripeptides are not synthesized because of weaker activity towards the alternative substrate in downstream activities, such as the condensation reaction.

Modules 2 and 3 of the *Nl* ACVS exhibit some degree of tolerance towards substrates that are structurally similar to the native ones, yielding significant levels of tripeptides. In marked contrast, module 1 accepted only three alternative substrates, L-Glu, DL-aminopimelic acid and 2-oxoadipic acid, resulting in the production of trace amounts of tripeptides. With L-Asp and DL-aminosuberic acid, no tripeptide could be detected. Considering the strong similarity of these structures with L-α-Aaa, it seems very clear that the length of the side chain is of crucial importance. The presence of the α-NH_2_ group appears to be crucial as well, as only trace amounts of tripeptide were detected in the reaction with 2-oxoadipic acid, while adipic acid was not incorporated at all (Table 1).

Importantly, in the structure of ACV, the peptide bond between the first two amino acids occurs between the δ-carboxyl group of L-α-Aaa and the amino group of L-Cys (Fig 1). Thus, L-α-Aaa must be adenylated on the side chain, in contrast with the canonical mechanism of activation of the C-α carboxyl group of amino acid described for other bacterial NRPSs [35][41]. Nonetheless, module 1 remains the most interesting target for potential engineering approaches, as the cysteine and valine are essential for β-lactam ring formation, while the aminoadipate is exchanged with other moieties in the (semi-) biosynthetic pathway of penicillins. Theoretically, by achieving activation and incorporation of alternative substrates at the first position, novel compounds with antibiotic activity could be generated.

Therefore, we proceeded with engineering the specificity of the adenylation domain of module 1. Several strategies have been used in the past to engineer NRPS enzymes with the goal of producing modified compounds, amongst which subdomain-, domain- and full module- exchanges, active site modification and directed evolution [33,42–47]. Herein, we designed a strategy based on the Golden Gate assembly method [34] and adapted from the work of Kries and coworkers on the Phe-specific GrsA initiation module of gramicidin S synthetase [33]. Using this strategy, we successfully generated 7 hybrid NRPS genes that could be expressed in *E*. *coli* HM0079 and purified. The peptide production assays and LC-MS analyses, however, revealed that only one of the hybrids was able to produce the expected tripeptide in an *in vitro* reaction, though at much lower levels. More specifically, the hybrid *Pc*A*Nl*CVS, with the same specificity as the native *Nl* ACVS, but with the subdomain region “implanted” from its *Penicillium chrysogenum* homologue.

While the difference in amino acid sequence between the fungal and bacterial ACVS was not a limiting factor, the narrow substrate range of the native enzyme seems to pose a greater obstacle to the engineering of functional NRPS hybrids with alternative specificities. Therefore, such engineering approaches could prove more successful when targeting naturally promiscuous enzymes. Recently it was reported that condensation domains also show specificity towards upstream activated substrate [48][49], and therefore exert an extra gate-keeping function. Thus, the chemistry of the ACVS reaction, the tight specificity shown by the first adenylation domain and its noncanonical interaction with the substrate, as well as the possibility of a second gate-keeping checkpoint on the condensation domain of module 2, all present significant challenges to the engineering of a functional hybrid ACVS capable of producing alternative tripeptides. Additionally, until the intra-NRPS reaction dynamics, conformational timing and structural organization of a multi-modular NRPS enzymatic system have been elucidated, global engineering efforts will remain challenging for this class of enzymes.

## Materials and methods

### Strains, plasmids and general culturing conditions

All cloning procedures were performed using *E*. *coli* DH5α. Cultures were grown using LB medium at 37 ^o^C and 200 rpm and antibiotic selection was conducted utilizing 25 μg/mL Zeocin. The *Nocardia lactamdurans pcbAB* was cloned using an intermediate gateway vector and was subsequently sub-cloned into the pBAD-plasmid (pBR322 ori; araC; pBAD, Zeo^R^) using SbfI x NdeI sites and including the introduction of a 6xHis-tag on the C-terminal end. This construct was kindly provided by DSM Sinochem Pharmaceuticals (now Centrient Pharmaceuticals). The synthetic DNA fragments encoding the donor subdomains were designed *in silico* and purchased from Invitrogen (GeneArt Strings). *In silico* PCR and cloning procedures, as well as subsequent analyses, were performed using the SnapGene® software (from GSL Biotech; available at snapgene.com).

### Identification of swapping partners and multi-alignment analysis

Donor NRPS were identified using the database NORINE [50]. For each of the substrate specificities that were selected for the engineering strategy (L-glutamic acid, L-aspartic acid, L-threonine, L-leucine and L-tyrosine), the database was searched using the function “monomer search”. The resulting hits were further selected based on sequence identity to the first adenylation domain of *Nl* ACVS, the size of the subdomains (determined according to the criteria described previously by Kries et al. [33]) and the identity between the putative donor subdomains and the L-α-aminoadipic acid-specific subdomain (S1 Fig). All alignment analyses were performed using the software MEGA7 [51] and Unipro UGENE [52]. The sequences of the complete adenylation domains from the donor NRPS were all aligned to the sequences of the subdomains designed and utilized by Kries and coworkers, to identify conserved motifs and define subdomain boundaries (Fig 5). All the DNA fragments were designed *in silico*, with the addition of the appropriate restriction sites, using the SnapGene® software. The DNA fragments were subsequently synthesized and purchased from Invitrogen (GeneArt Strings). The L-Val specific subdomain from *Nl* ACVS M3 and the L-α-Aaa-specific subdomain from *Pc* ACVS M1 were amplified via PCR from pBAD-*Nl* ACVS and *P*. *chrysogenum* DS47274 gDNA, respectively. KAPA HiFi HotStart ReadyMix (Roche) was used, according to the manufacturer’s protocol.

### Engineering of the first adenylation domain of *Nl* ACVS via a Golden Gate-based subdomain swap strategy

The pBAD plasmid containing the gene encoding *Nl* ACVS was virtually divided in three fragments, called A1, A2 and A3, designed *in silico* in such a way to exclude the *Nl* ACVS M1 native subdomain (S2 Fig). The three fragments were then individually amplified via PCR using KAPA HiFi HotStart ReadyMix (Roche), according to the manufacturer’s protocol, and sub-cloned into intermediate pMAL-c5x-BsrDI free vectors (mutated in house to remove the recognition site BsrDI, type IIs restriction enzyme used in the Golden Gate assembly). The DNA fragments encoding the selected subdomains were sub-cloned in the same intermediate vector. The constructs were then checked via restriction analysis and sequencing (Macrogen Inc.). Once all the parts were confirmed correct, Golden Gate assembly reactions were performed to build the hybrid NRPSs. For this, the intermediate vectors pMAL-c5x-A1, pMAL-c5x-A2, pMAL-c5x-A3 and pMAL-c5x-sub_x_ were mixed with a molar ratio of 1:1:0.5:1, with a total amount of DNA ~ 500 ng. Subsequently, 1 μL of T4 DNA Ligase (5 U/μL), 1 μL of T4 DNA Ligase 10x buffer and 0.5 μL of BsrDI (5 U/μL) (Invitrogen, Thermo Fisher Scientific) were added to complete the reaction mixture in a total volume of 10 μL. The Golden Gate assembly reactions were carried out in a C1000 Thermal Cycler (Bio-Rad), with the standard 50x cycles-protocol [34]. The reaction mixtures were directly transformed into chemically-competent *E*. *coli* DH5α cells. The plasmids were subsequently mini-prepped and checked as described previously.

### Expression and His-tag affinity purification of *Nl* ACVS and hybrid NRPSs

Cultures were grown to an OD_600_ of 0.6, transferred to 18°C and 200 rpm for 1h and subsequently induced using 0.2% L-arabinose. Harvest followed 18h after induction by spinning at 4000 g for 15 minutes. After resuspension in lysis buffer (HEPES 50 mM pH 7.0, 300 mM NaCl, 2 mM DTT, Complete EDTA free protease inhibitor; Roche No. 04693159001), cells were disrupted using sonication (6s/15s on/off; 50x; 10μm amplitude; Soniprep 150 MSE) and cell-free lysate obtained by centrifugation at 4°C, 17000g, 15 minutes. Purified enzyme was extracted by means of Ni^2+^ affinity purification using gravity flow. Wash steps were performed using two column volumes of wash buffer (HEPES 50 mM pH 7.0, 300 mM NaCl, 20 mM imidazole) followed by a three-step elution using one bed volume of each elution buffer (HEPES 50 mM pH7.0, NaCl 300 mM, imidazole 50–150 or 250 mM). Samples were concentrated if necessary, using Amicon U-100 spin filters (Merck). Final concentration was determined using A_280_ (NanoDrop 1000; Thermo Fisher Scientific).

### *In vitro* product formation assay

Isolated enzymes (fraction E3) were subjected to *in vitro* assays, in order to determine product formation properties. Assay conditions initially used include HEPES 50 mM pH 7.0, 300 mM NaCl, 5 mM ATP pH 7.0, 100 μM CoA, 0.2 μM phosphopantetheinyl transferase (Sfp, NEB), 5 mM L-α-aminoadipic acid, 2 mM L-cysteine, 2 mM L-valine (5 mM L-Val for the hybrid VCVS), 5 mM MgCl_2_, 2 mM DTT and 0.17 μM ACVS, or 0.5–1 μM for the hybrids. For velocity and affinity determination, amino acid concentration of 0.1, 0.25, 0.5, 1, 2 and 5 mM were used. For the hybrids’ reactions and promiscuity determination the concentration of the variable amino acid was set at 5mM. Reactions were run at 30°C and sampling took place after 0, 10, 20, 30, 45, 60, 120 and 240 minutes for dynamic measurements and after 0 and 240 or 960 minutes for endpoint value determination. NaOH was added to each sample to a final concentration of 0.1 M to quench the reactions. Samples were subsequently stored at -80°C and reduced before HPLC/MS analysis adding DTT to a concentration of 5 mM.

### High performance liquid chromatographic and mass-spectrometric analysis (HPLC/MS)

Samples (50 μL) obtained from an *in vitro* reaction were subjected to HPLC/MS analysis. Two technical replicates were run per sample at 5 μL each. Analysis was performed using a LC/MS Orbitrap (Thermo Scientific) in combination with a RP-C18 column (Shimadzu Shim pack XR-ODS 2.2; 3.0x75mm). Scan range was set at 80–1600 M/Z in positive ion (4.2kV spray, 87.5V capillary and 120V of tube lens) mode, with capillary temperature set at 325°C. A gradient program with MilliQ water (A), Acetonitrile (B) and 2% Formic acid (C) was run: 0 min, A 90%, B 5%, C 5%; 4 min, A 90%, B 5%, C 5%; 13 min, A 0%, B 95%, C 5%; 16 min, A 0%, B 95%, C 5%; 16 min, A 90%, B 5%, C 5%; 21 min, A 90%, B 5%, C 5% at a flow rate of 0.3 ml min^-1^. The Bis-ACV standard was obtained from Bachem, reduced to (L,L,D)-ACV and used for quantification in a standard curve at concentrations of 0.1, 0.5, 1, 5, 10, 50 and 100 μM. Alternative tripeptides were identified according to accurate monoisotopic mass, if not mentioned otherwise.

## Supporting information

S1 Fig Selection criteria for donor subdomain templates. The donor subdomains were selected according to three criteria. First, we individually aligned the full donor A domains to the ACVS M1 A domain (L-Aaa) and determined the sequence identity (we selected those with identity higher than 30% for further analysis, in green). We then determined the size of the subdomain, using as boundaries the regions described in the methods section and Fig 5; those with a similar size to the wild-type subdomain were aligned with the latter, to determine the identity between the subdomains themselves. The ones with highest identity were selected and designed *in silico* for the assembly strategy (highlighted in dark green). Targets with A domains sequence identities below 30% were not further included in the analyses; *local misalignments that prevented the determination of the subdomains boundaries.
(TIF)

S2 Fig Golden gate-based subdomain swap strategy. (**A**) pBAD-*Nl* ACVS His-tag plasmid map (exported from Snapgene). (**B**) Hybrid NRPSs assembly strategy: three fragments (named A1, A2 and A3) were amplified via PCR from the plasmid in such a way to amplify the gene together with the vector and exclude the subdomain of module 1; the three fragments were cloned into pMAL-c5x-BsrDI free intermediate vectors (BsrDI sites are presents at the ends of the A1, A2 and A3 fragments for the Golden Gate assembly); the synthetic donor subdomains (Asub_x_) are also cloned into the same intermediate vector. A1, A2, A3 and Asub_x_ have complementary overhangs (indicated by numbers 1–4) after digestion with BsrDI, allowing the Golden Gate assembly reaction.
(TIF)

S3 Fig Predicted structures of hybrid tripeptides. The structures (wild-type product ACV on top) were drawn using MarvinSketch (ChemAxon), and exact molecular weights were determined using the ‘*Elemental analysis*’ tool of the same software.
(TIF)

S1 Appendix LC chromatograms and mass spectra of characterized tripeptides. The full chromatograms were filtered in accordance with the predicted m/z value of each tripeptide. The mass spectra of the resulting peaks were scanned for the presence of the expected compound.
(PDF)

S1 Raw Images (PDF)
