# Supplementary figures and images for: Biochemical characterization of the Nocardia lactamdurans ACV synthetase
Source: PLoS One. 2020 Apr 10;15(4):e0231290. doi: 10.1371/journal.pone.0231290 (PMC7147772; doi:10.1371/journal.pone.0231290)

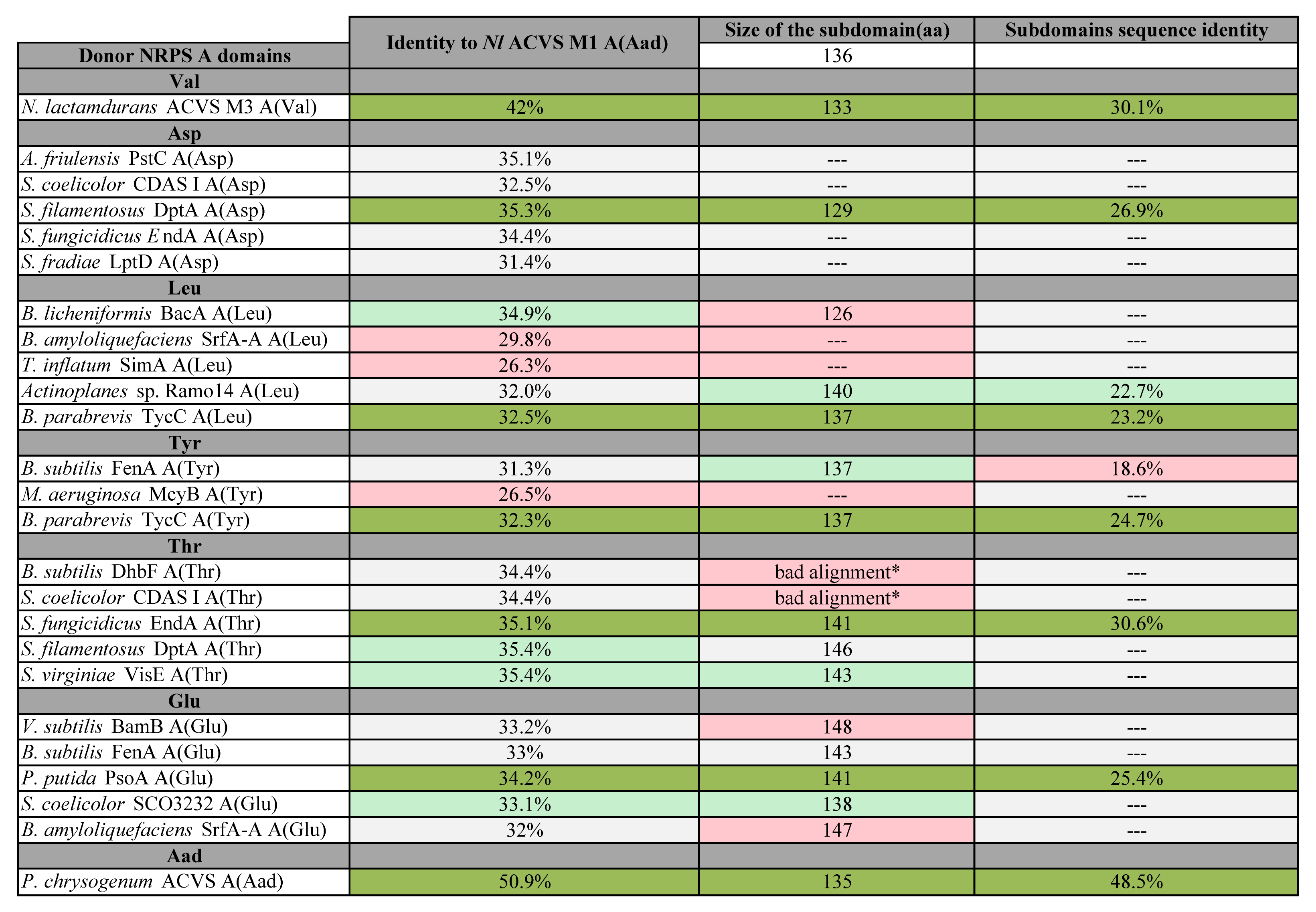

Supplement: S1 Fig — The donor subdomains were selected according to three criteria. First, we individually aligned the full donor A domains to the ACVS M1 A domain (L-Aaa) and determined the sequence identity (we selected those with identity higher than 30% for further analysis, in green). We then determined the size of the subdomain, using as boundaries the regions described in the methods section and Fig 5; those with a similar size to the wild-type subdomain were aligned with the latter, to determine the identity between the subdomains themselves. The ones with highest identity were selected and designed in silico for the assembly strategy (highlighted in dark green). Targets with A domains sequence identities below 30% were not further included in the analyses; *local misalignments that prevented the determination of the subdomains boundaries. (TIF) [file pone.0231290.s001.tif]

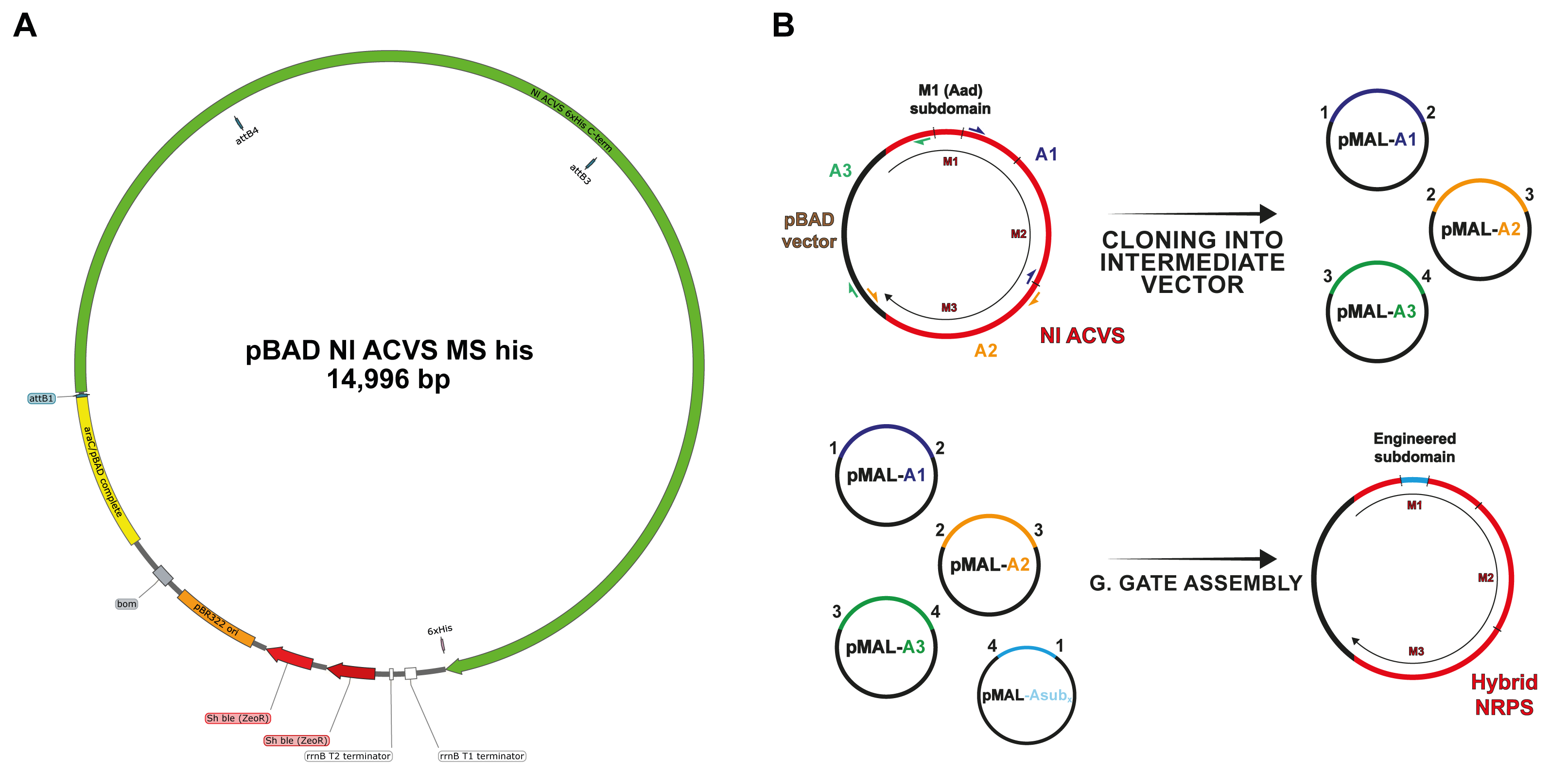

Supplement: S2 Fig — (A) pBAD-Nl ACVS His-tag plasmid map (exported from Snapgene). (B) Hybrid NRPSs assembly strategy: three fragments (named A1, A2 and A3) were amplified via PCR from the plasmid in such a way to amplify the gene together with the vector and exclude the subdomain of module 1; the three fragments were cloned into pMAL-c5x-BsrDI free intermediate vectors (BsrDI sites are presents at the ends of the A1, A2 and A3 fragments for the Golden Gate assembly); the synthetic donor subdomains (Asubx) are also cloned into the same intermediate vector. A1, A2, A3 and Asubx have complementary overhangs (indicated by numbers 1–4) after digestion with BsrDI, allowing the Golden Gate assembly reaction. (TIF) [file pone.0231290.s002.tif]

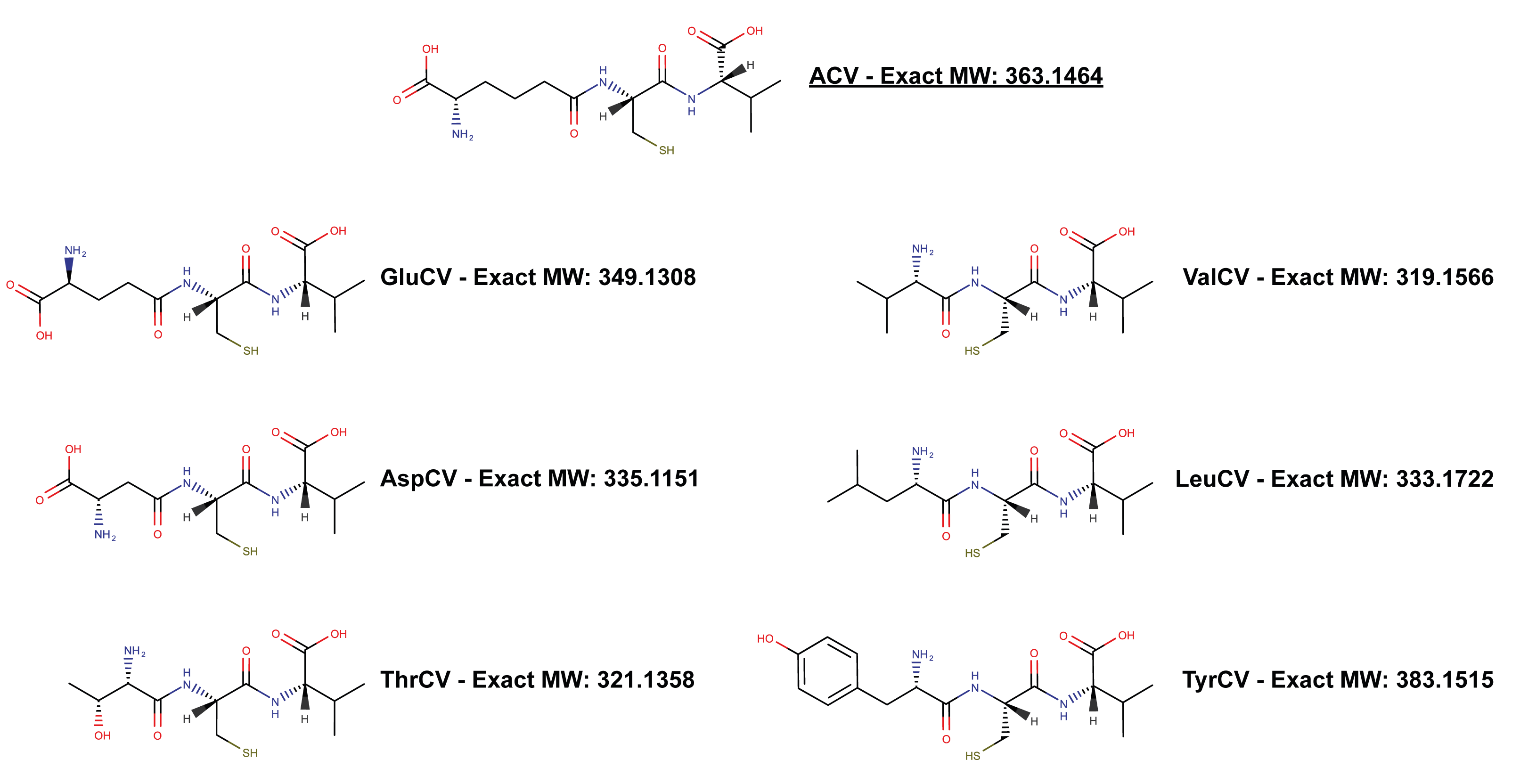

Supplement: S3 Fig — The structures (wild-type product ACV on top) were drawn using MarvinSketch (ChemAxon), and exact molecular weights were determined using the ‘Elemental analysis’ tool of the same software. (TIF) [file pone.0231290.s003.tif]
