# Supplementary material for: Biochemical characterization of the Nocardia lactamdurans ACV synthetase
Source: PLoS One. 2020 Apr 10;15(4):e0231290. doi: 10.1371/journal.pone.0231290 (PMC7147772; doi:10.1371/journal.pone.0231290)

ACVS reaction - L-α-Aaa, L-Cys, L-Val (native substrates)
Expected m/z of ACV tripeptide: 363.146 (M_i) + 1.008 (H^+) = 364.154

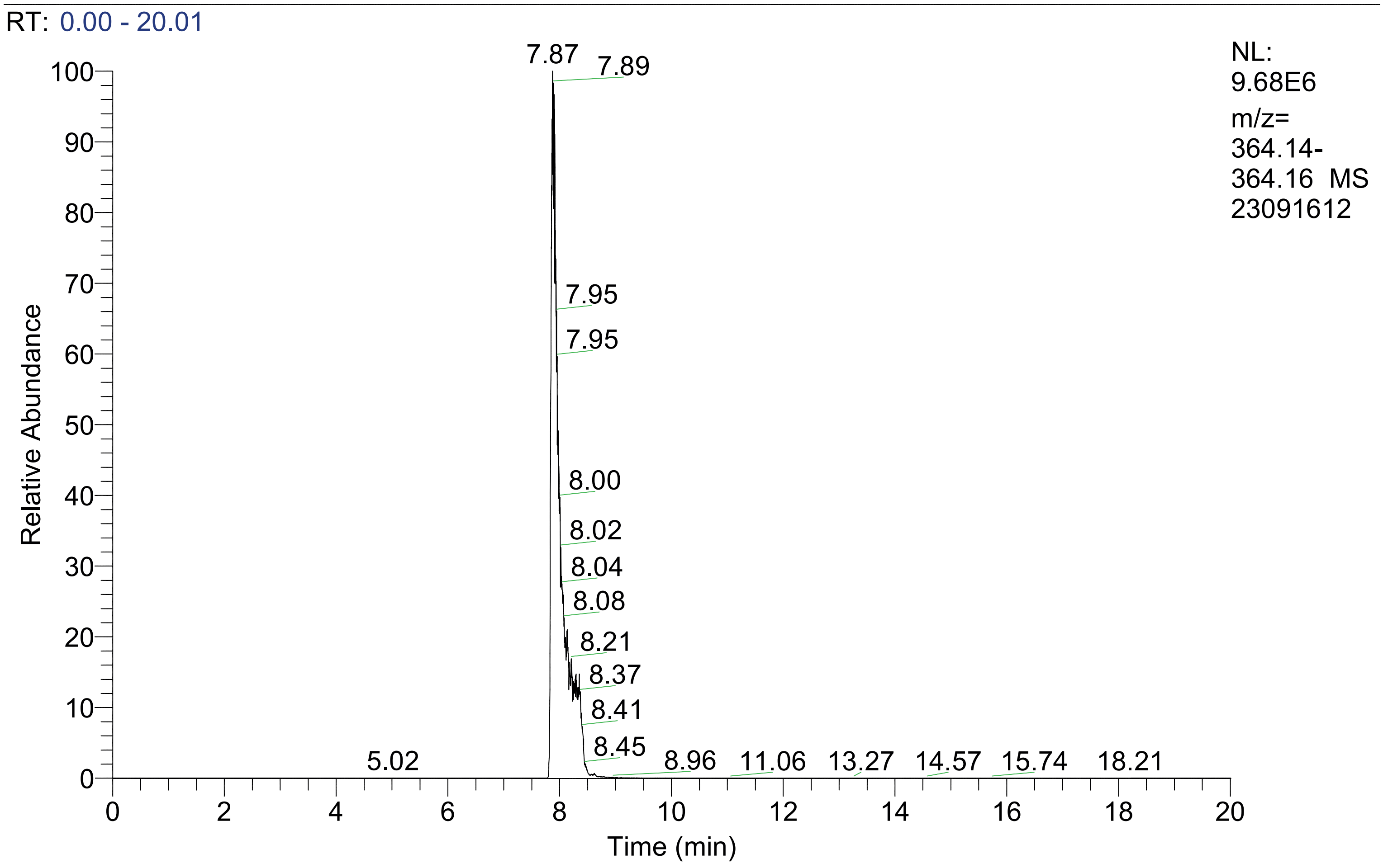

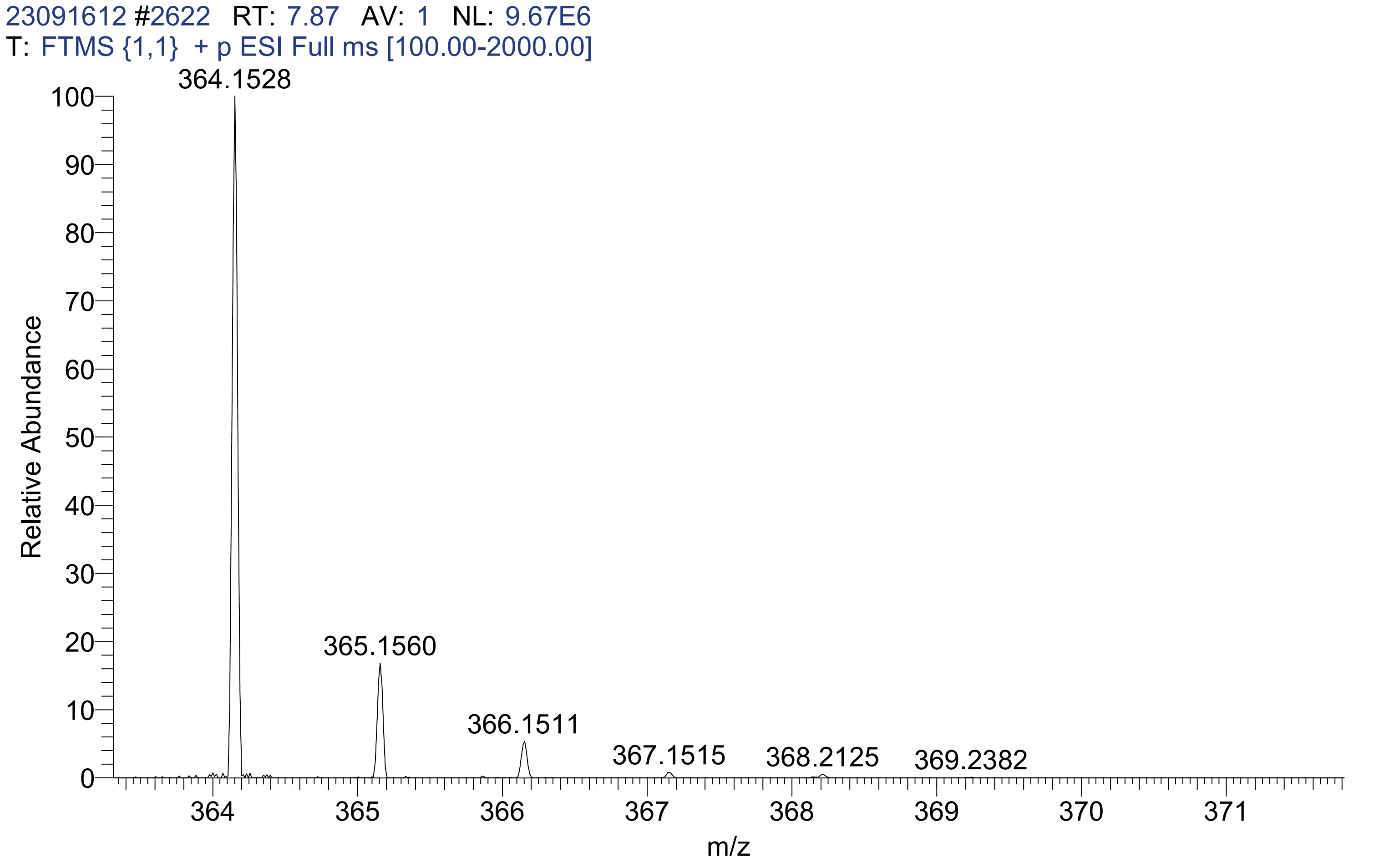

ACVS reaction - substrates: L-Glu, L-Cys, L-Val
Expected m/z of Glu-CV tripeptide: 349.131 (M_i) + 1.008 (H^+) = 350.139

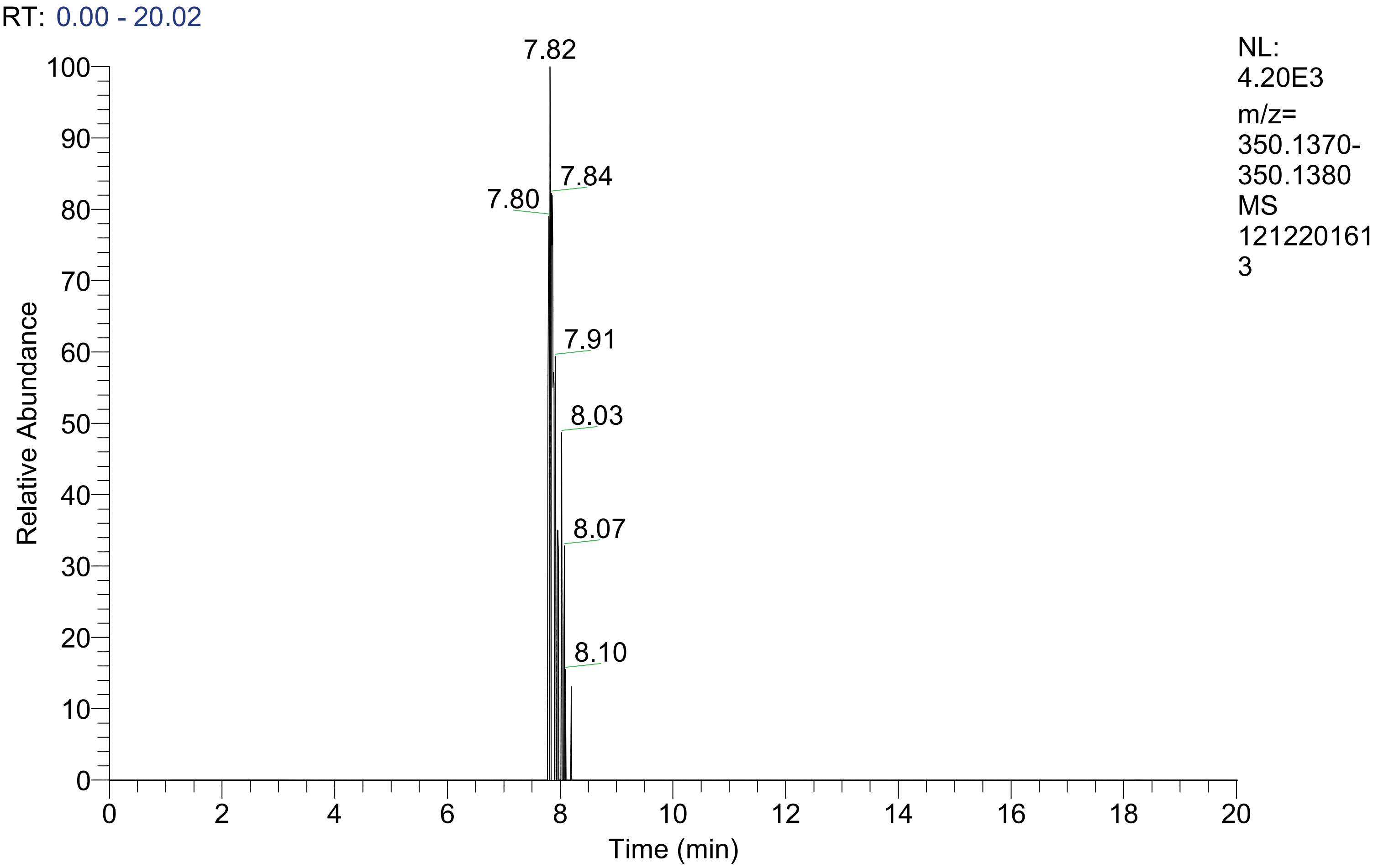

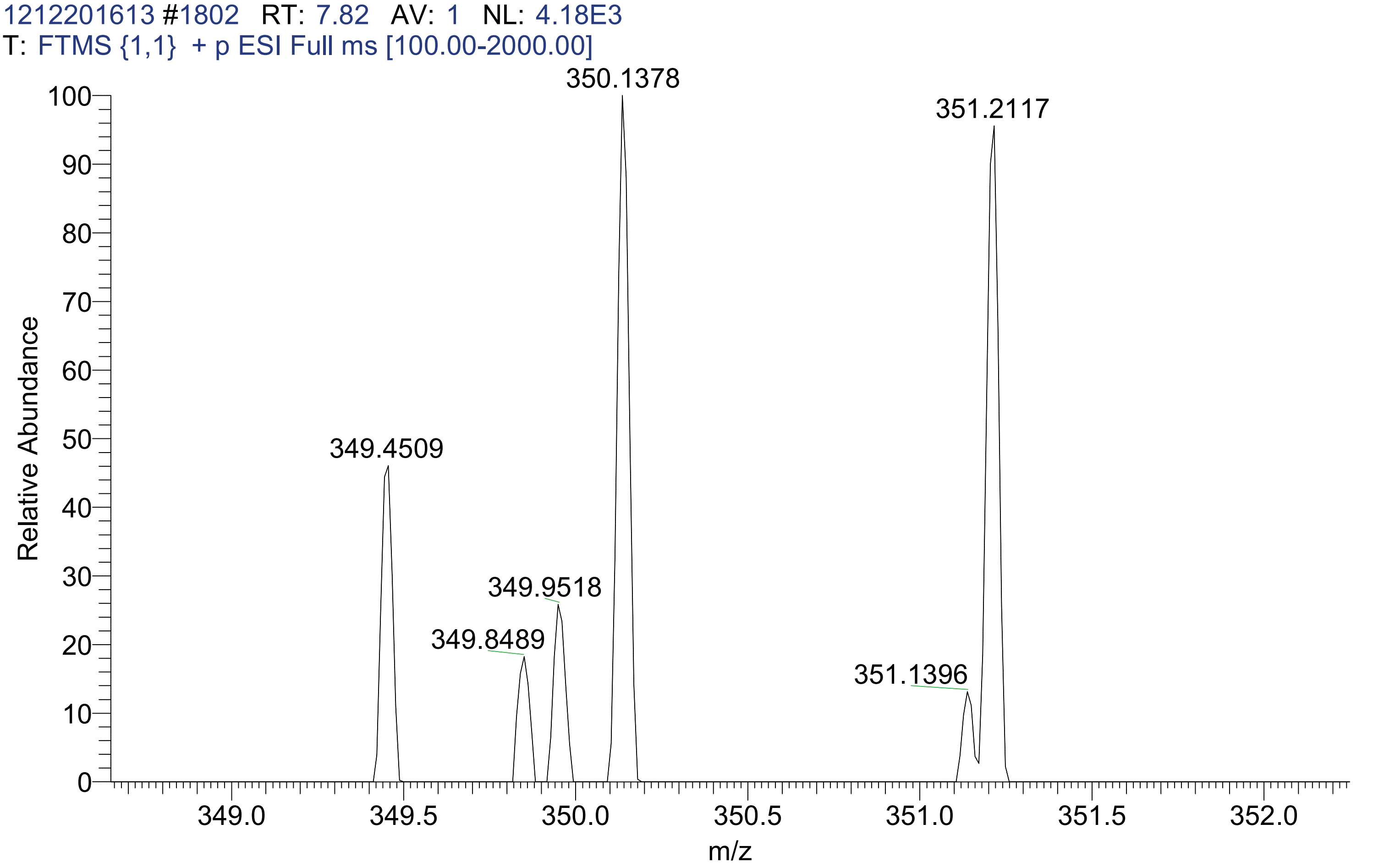

ACVS reaction - substrates: DL-aminopimelic acid, L-Cys, L-Val
Expected m/z of API-CV tripeptide: 377.162 (M_i) + 1.008 (H^+) = 378.170

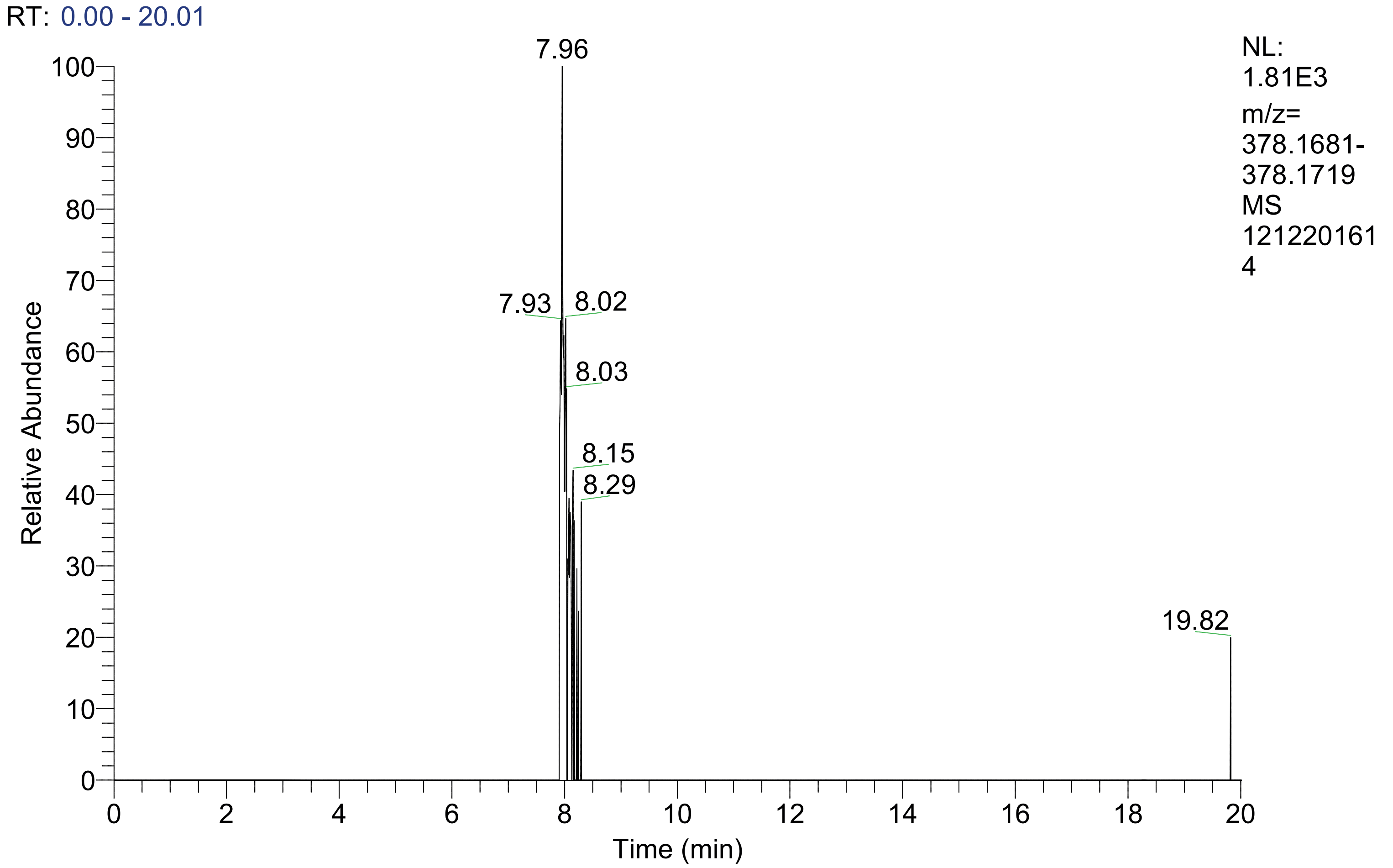

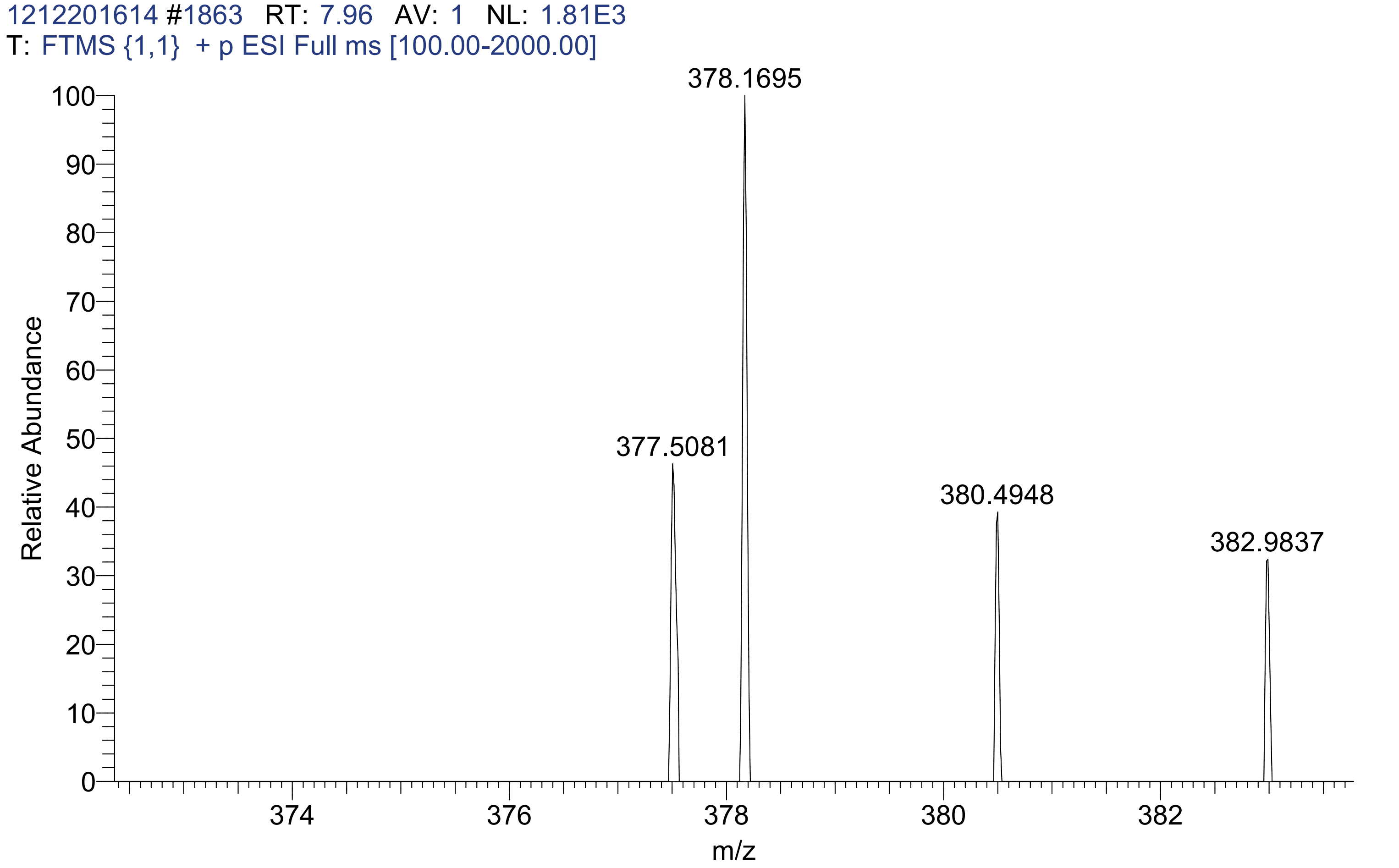

ACVS reaction - substrates: 2-oxoadipic acid, L-Cys, L-Val
Expected m/z of OAA-CV tripeptide: 362.115 (M_i) + 1.008 (H^+) = 363.123
***24 minutes LC method (same gradient)**

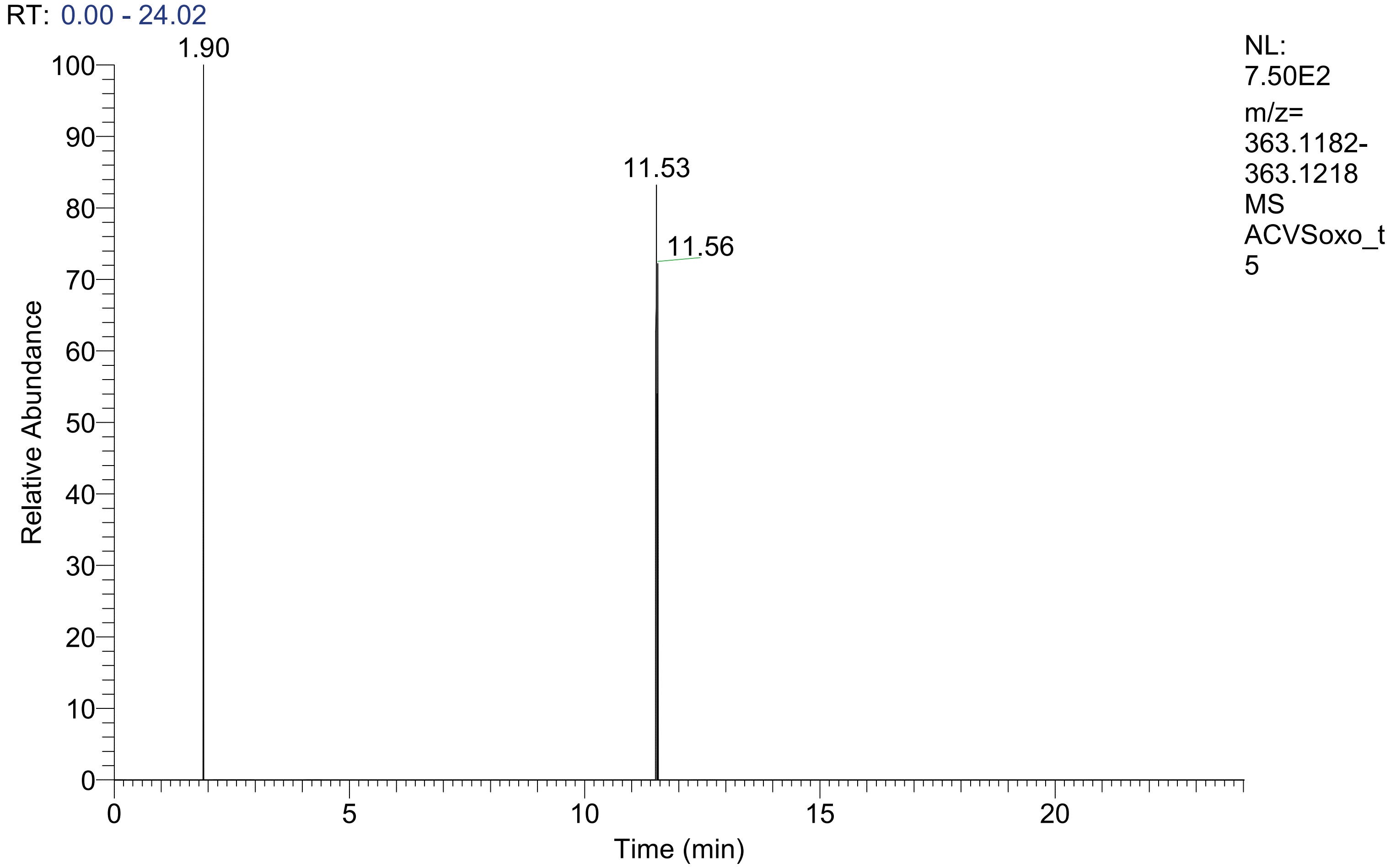

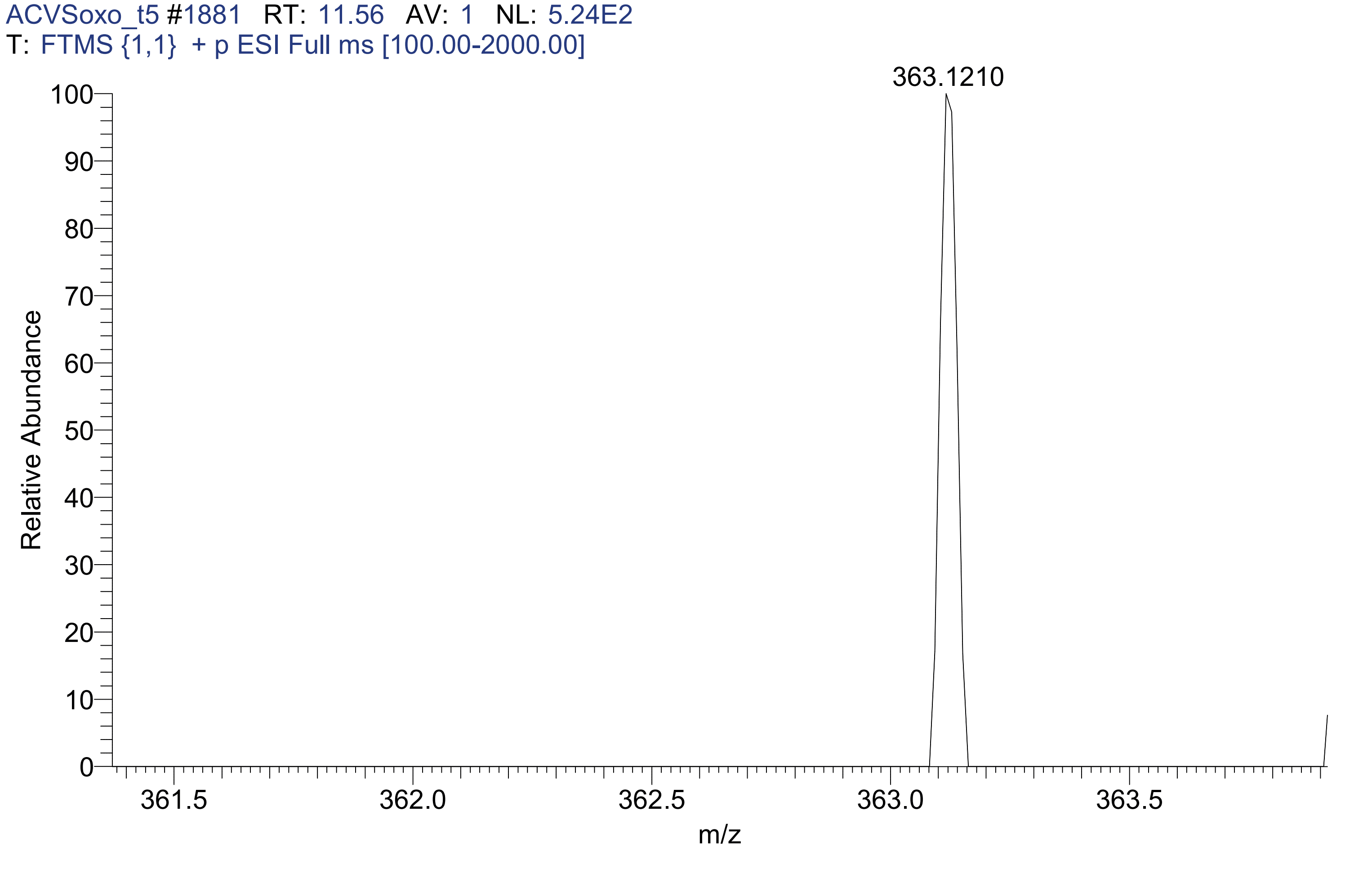

ACVS reaction - substrates: L-α-Aaa, L-Ala, L-Val
Expected m/z of A-Ala-V tripeptide: 331.174 (M_i) + 1.008 (H^+) = 332.182

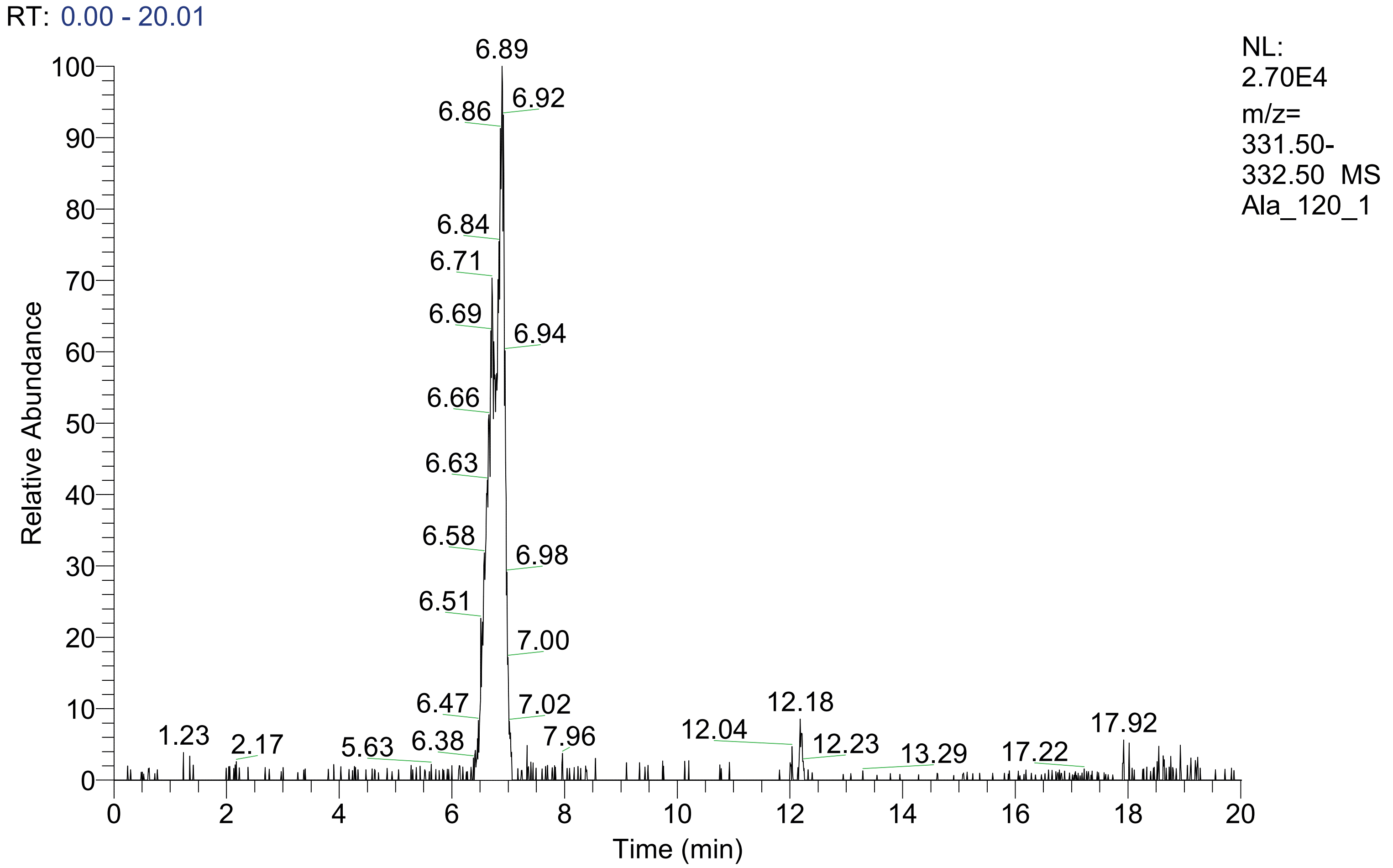

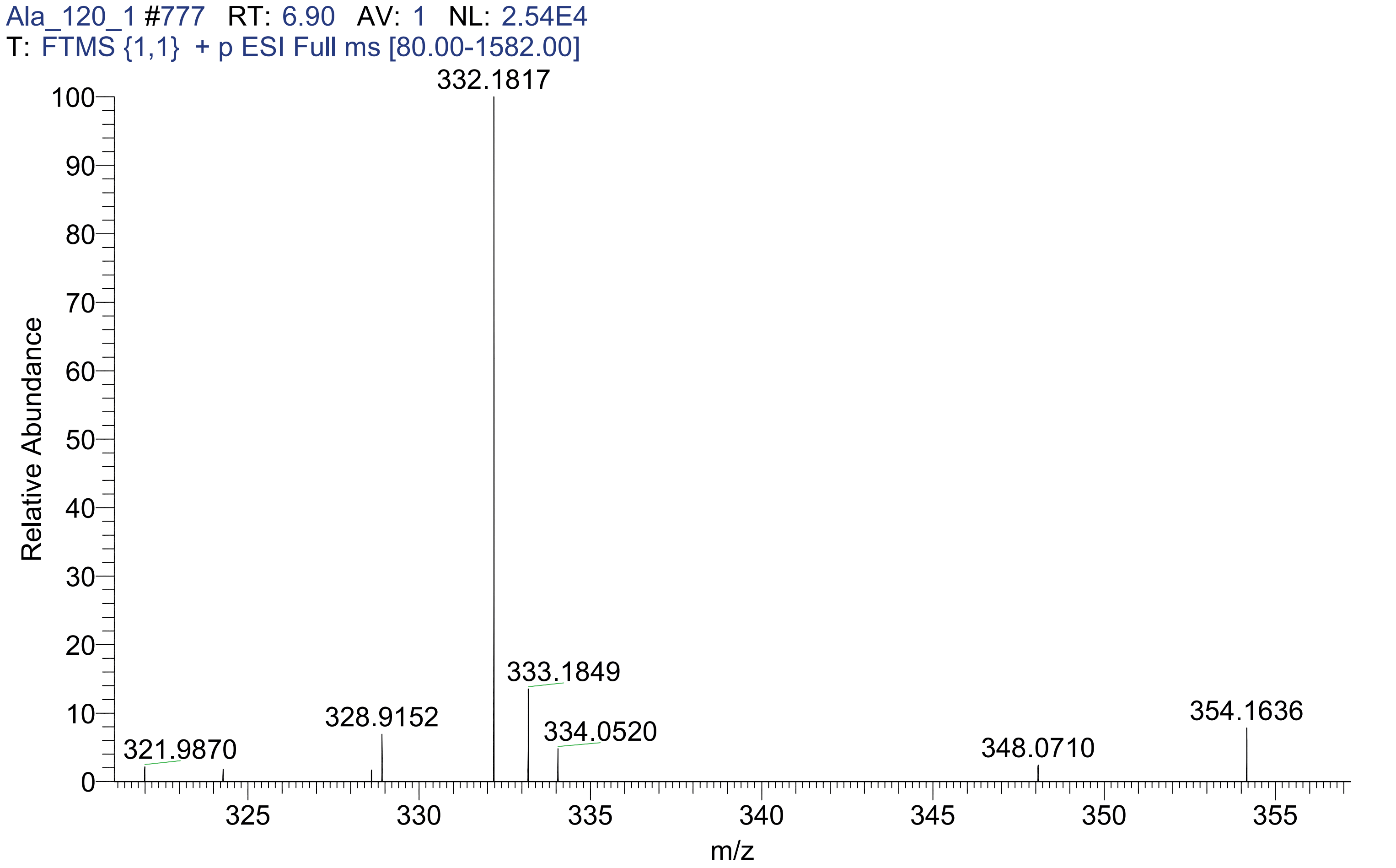

ACVS reaction - substrates: L-α-Aaa, L-Thr, L-Val
Expected m/z of A-Thr-V tripeptide: 361.185 (M_i) + 1.008 (H^+) = 362.193

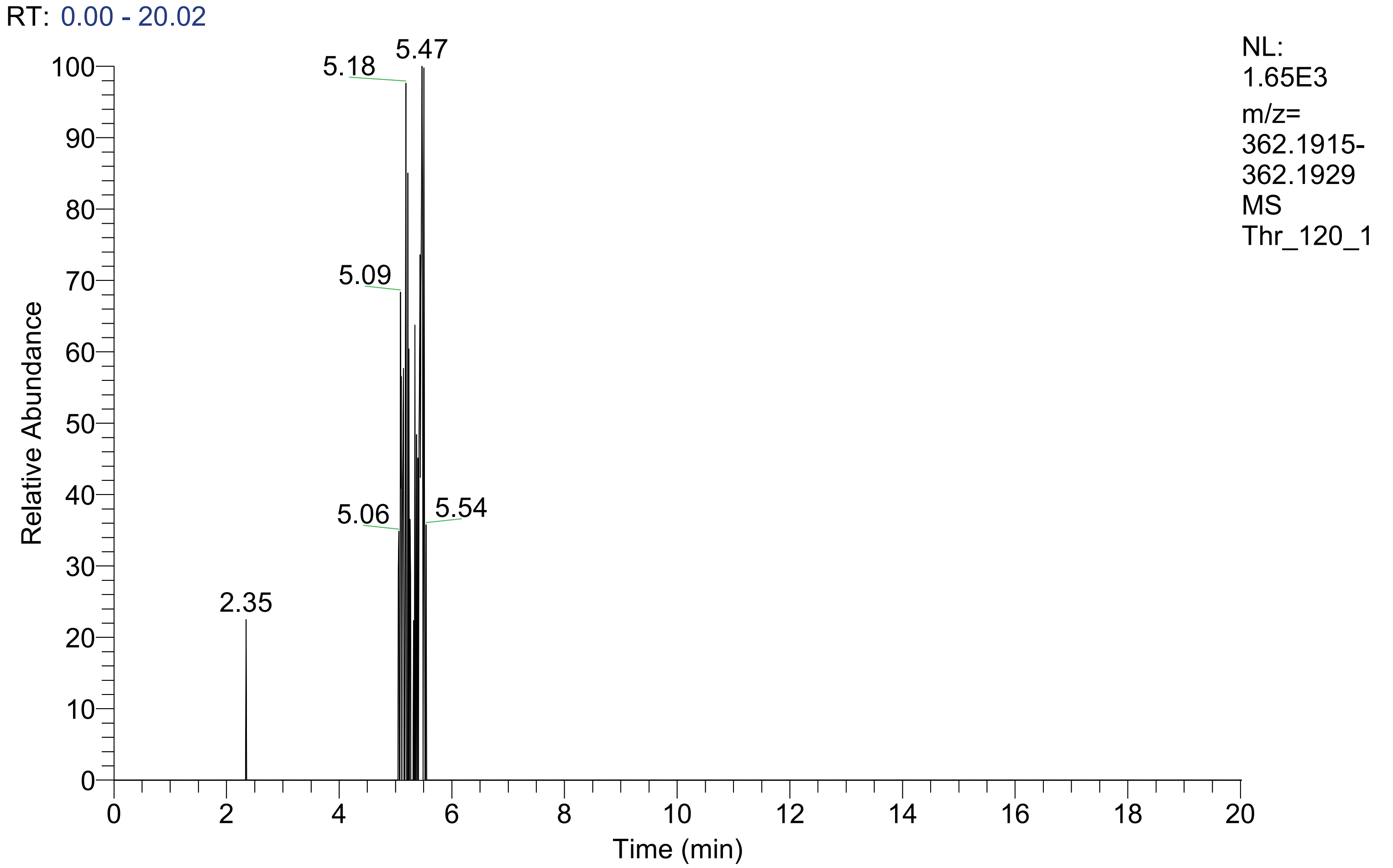

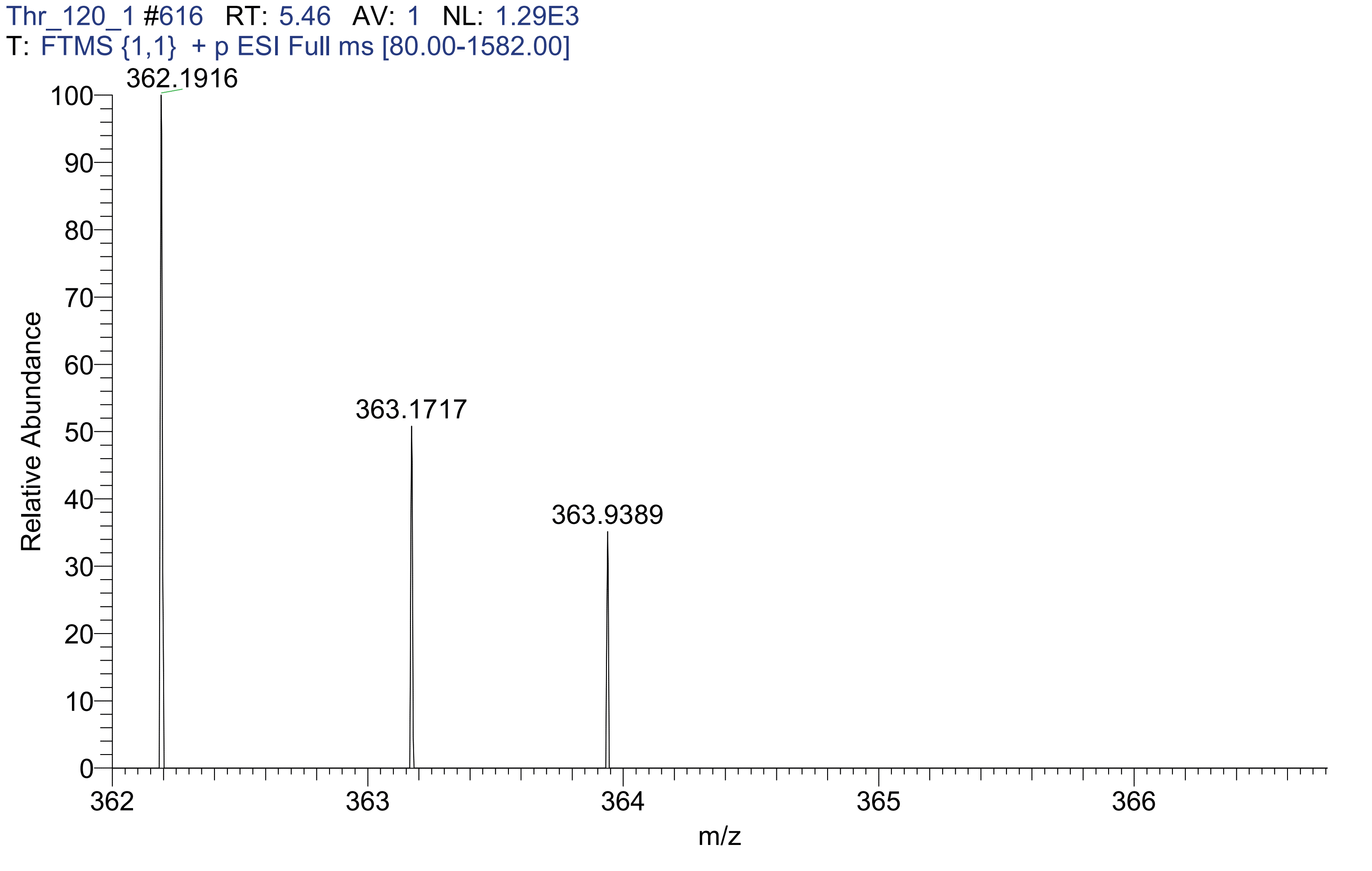

ACVS reaction - substrates: L-α-Aaa, L-Penicillamine, L-Val
Expected m/z of A-Pen-V tripeptide: 391.178 (M_i) + 1.008 (H^+) = 392.186

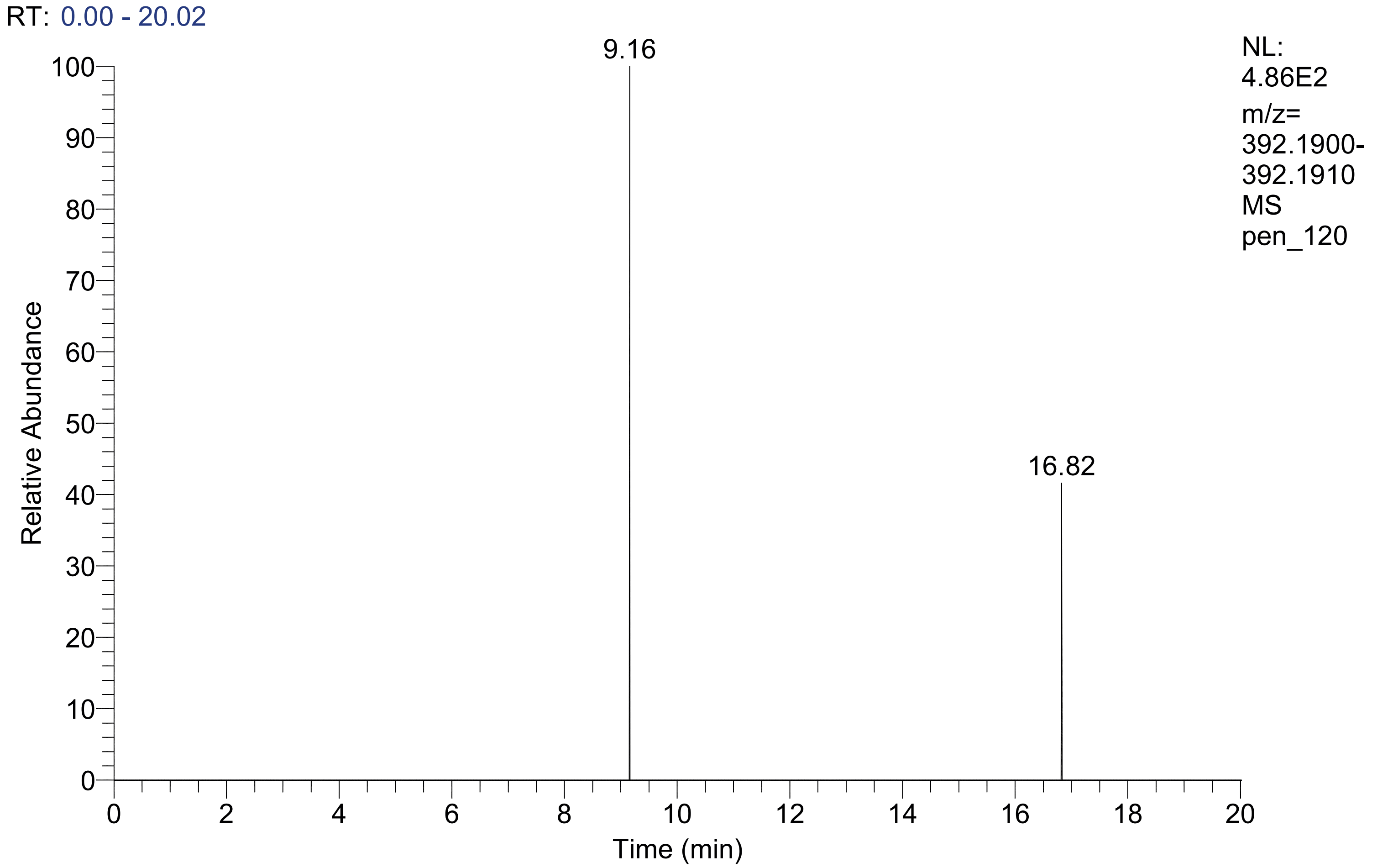

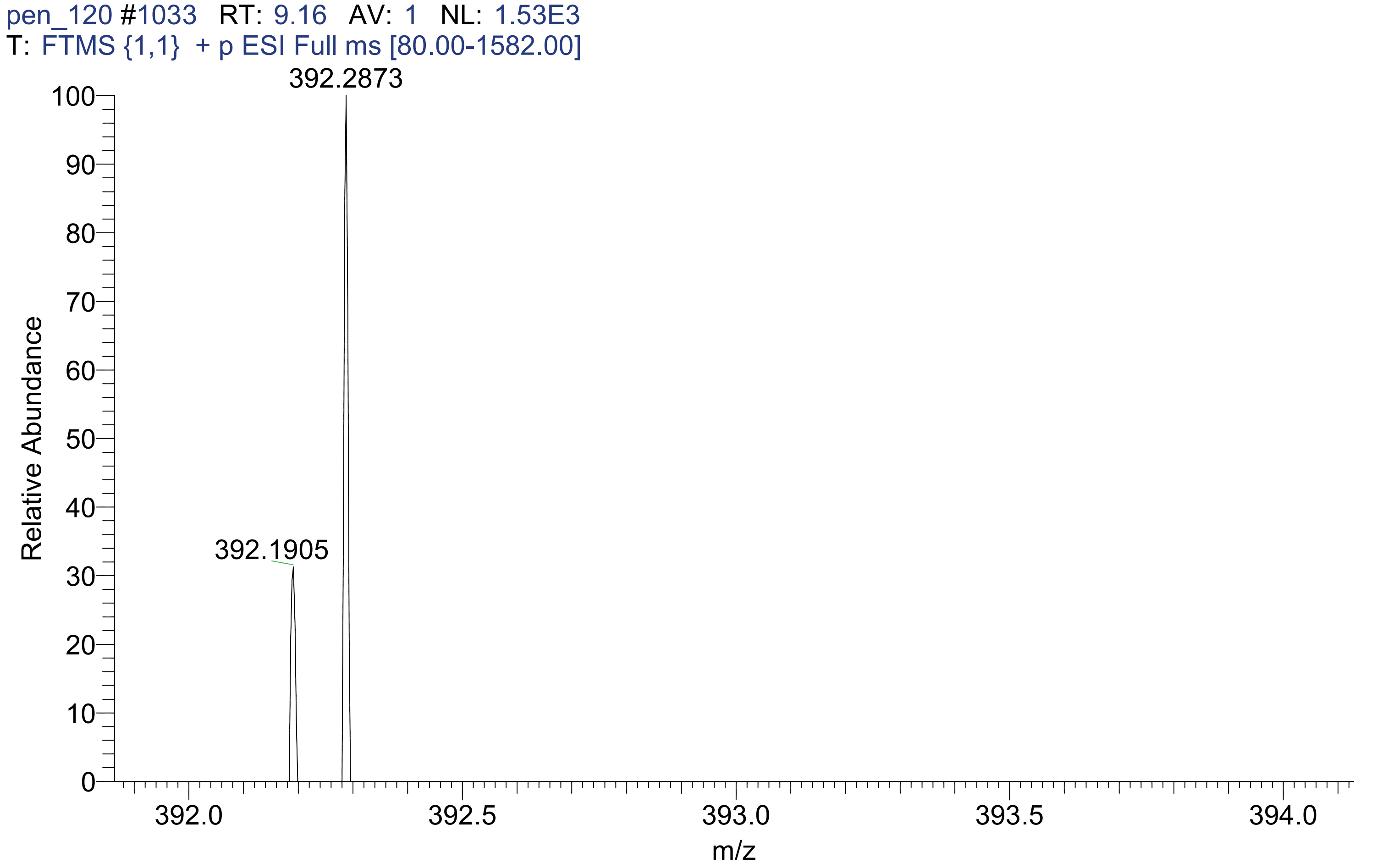

ACVS reaction - substrates: L-α-Aaa, L-Methionine, L-Val
Expected m/z of A-Met-V tripeptide: 391.178 (M_i) + 1.008 (H^+) = 392.186

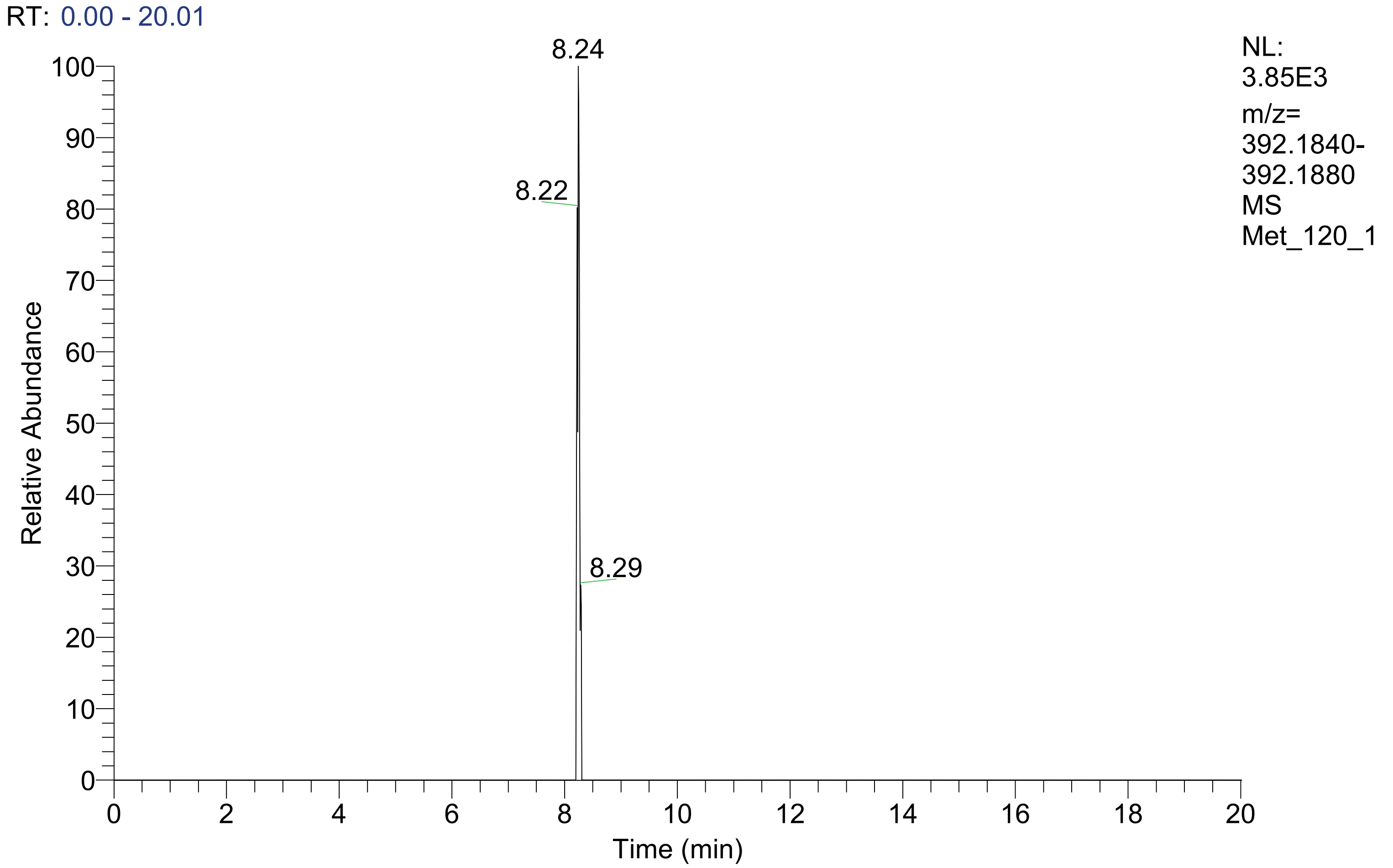

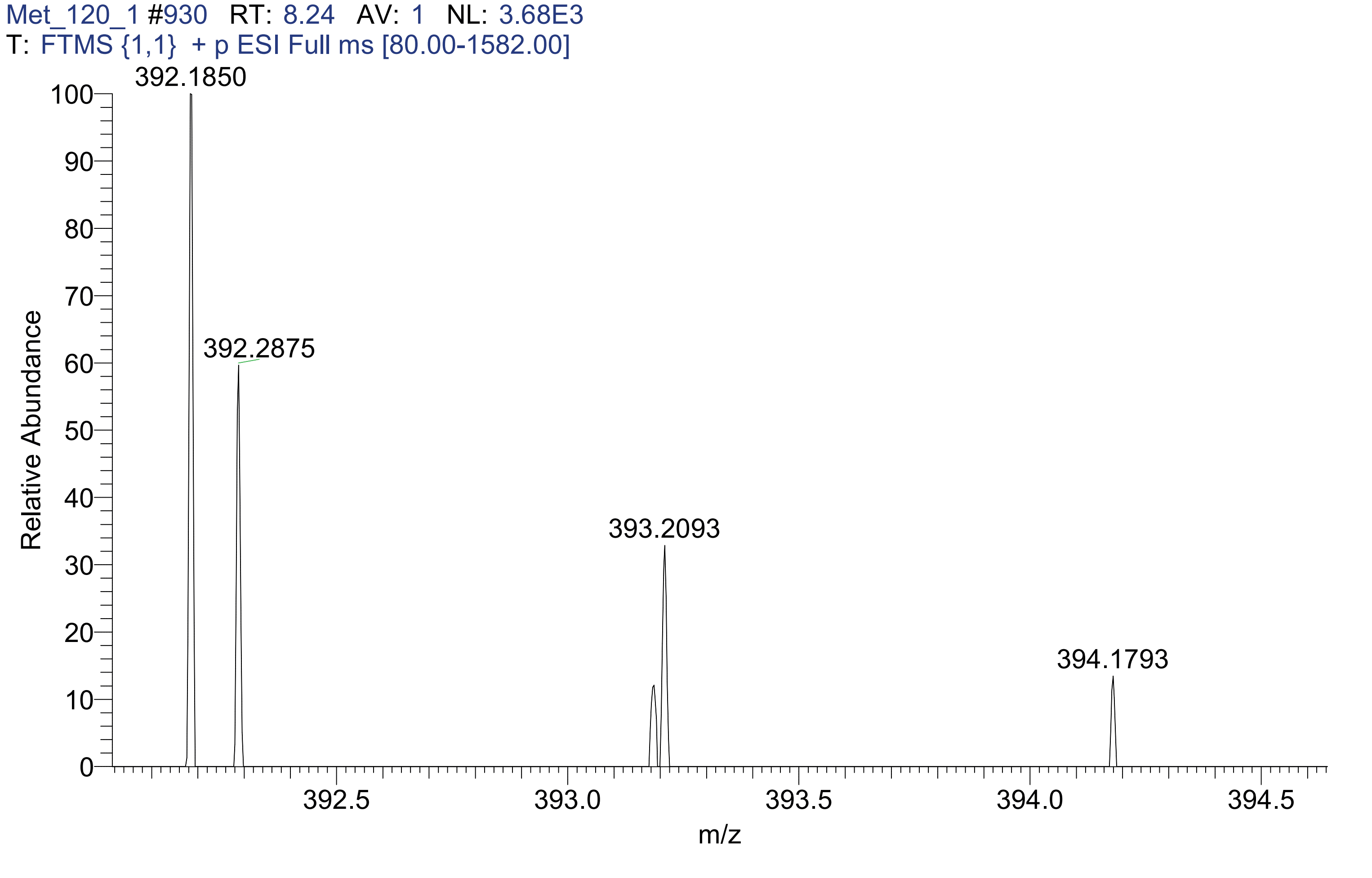

ACVS reaction - substrates: L-α-Aaa, L-Leucine, L-Val
Expected m/z of A-Leu-V tripeptide: 373.221 (M_i) + 1.008 (H^+) = 374.229

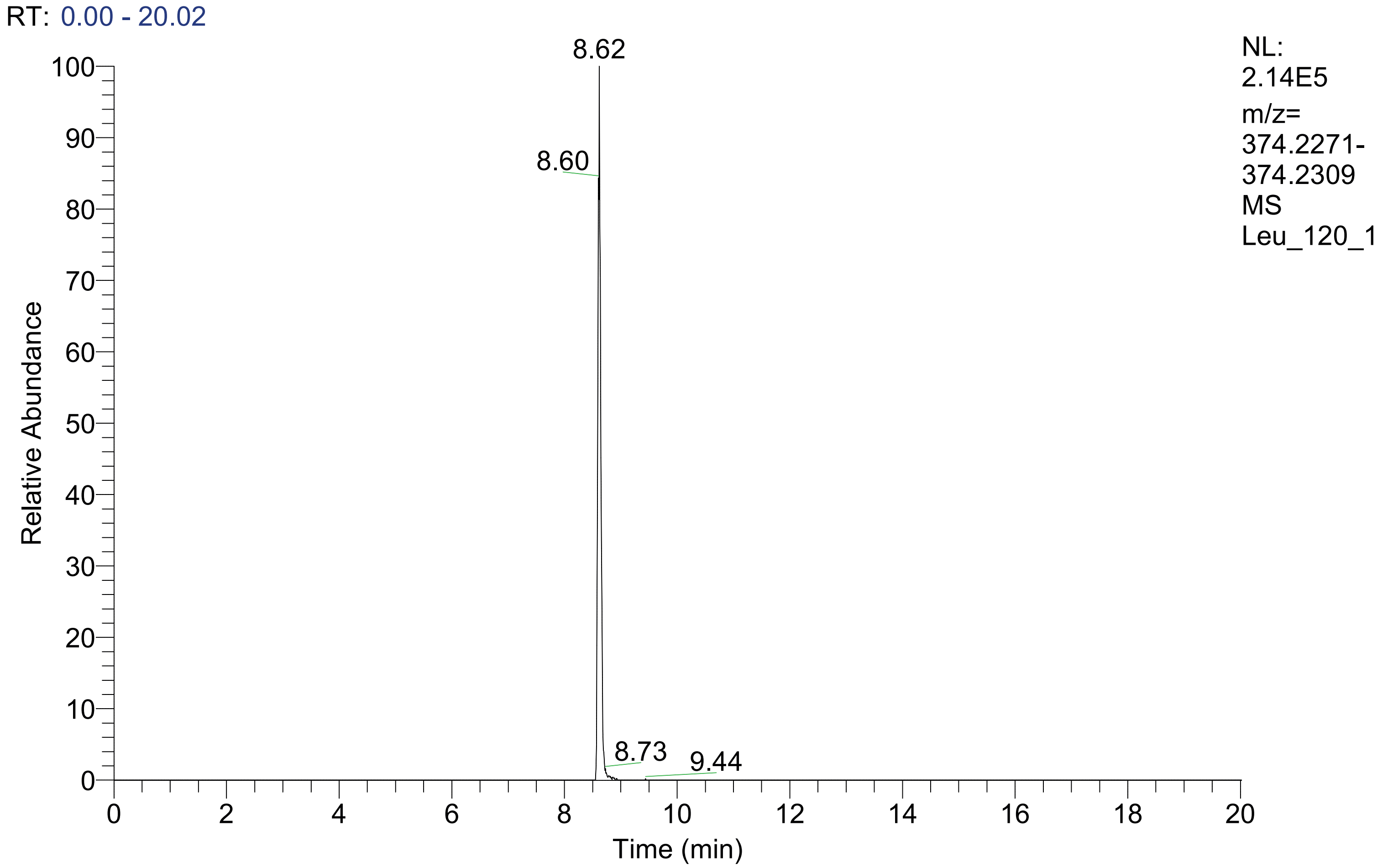

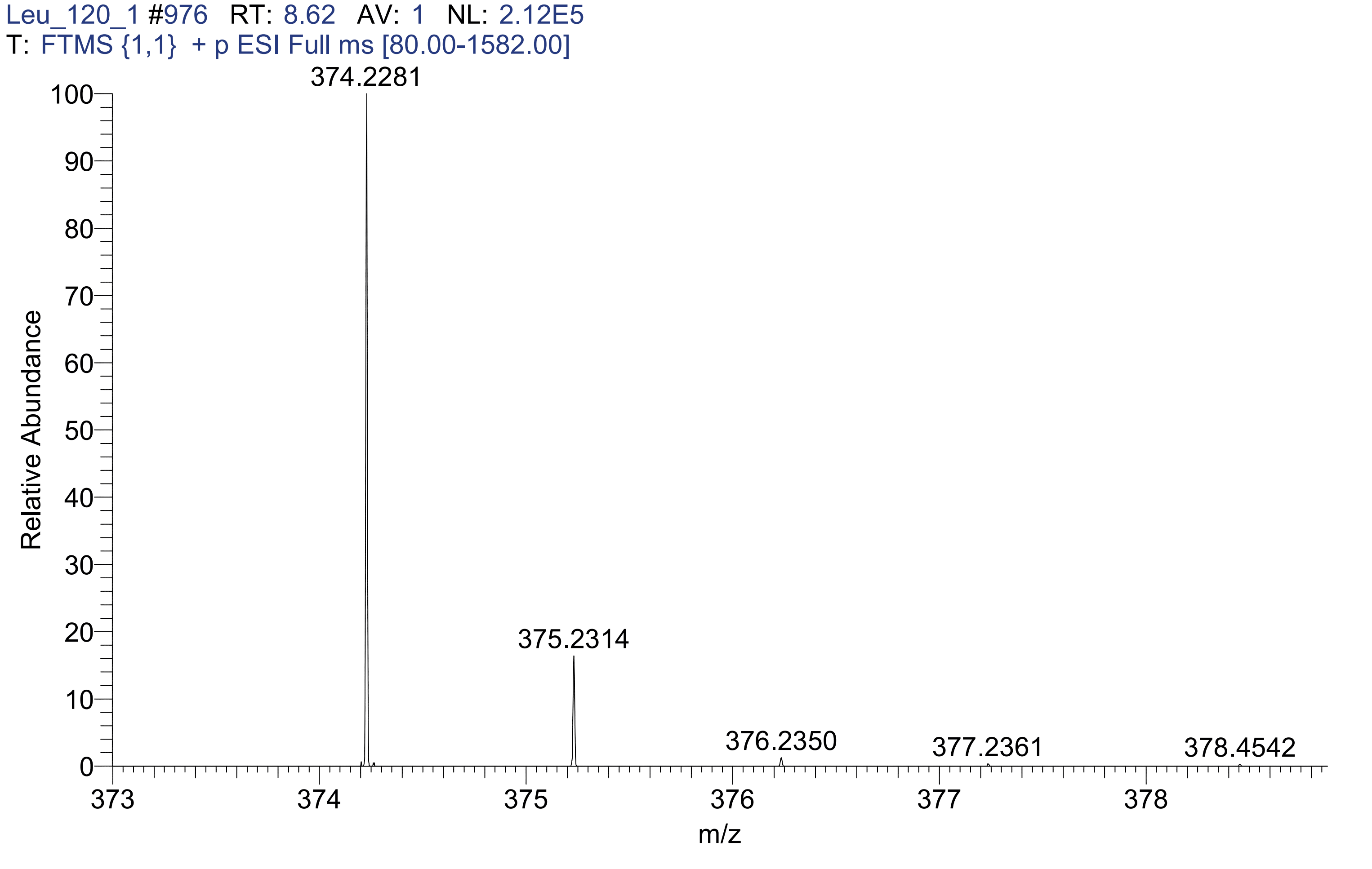

ACVS reaction - substrates: L-α-Aaa, L-Cys, L-Norvaline
Expected m/z of AC-Norval tripeptide: 363.146 (M_i) + 1.008 (H^+) = 364.154

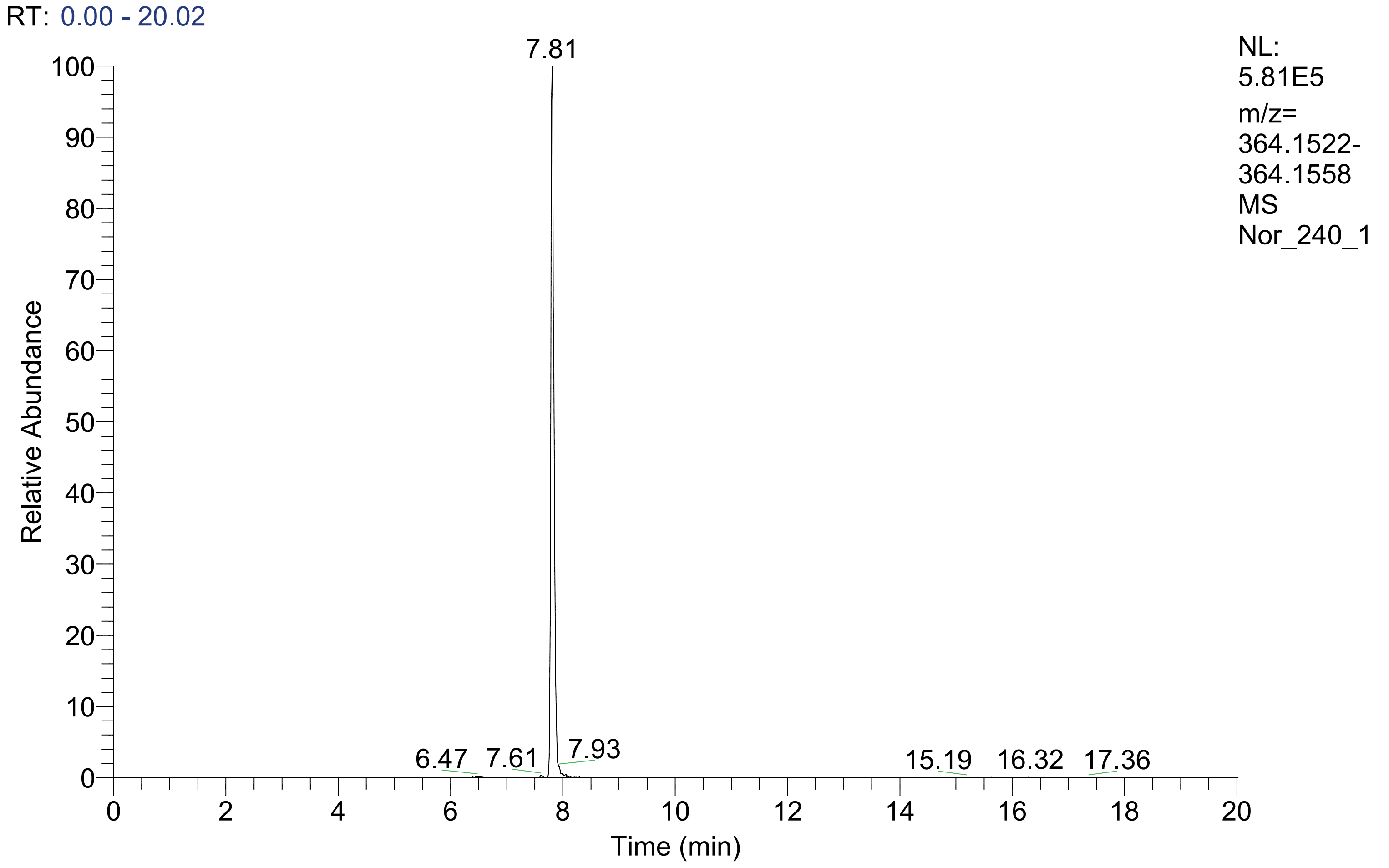

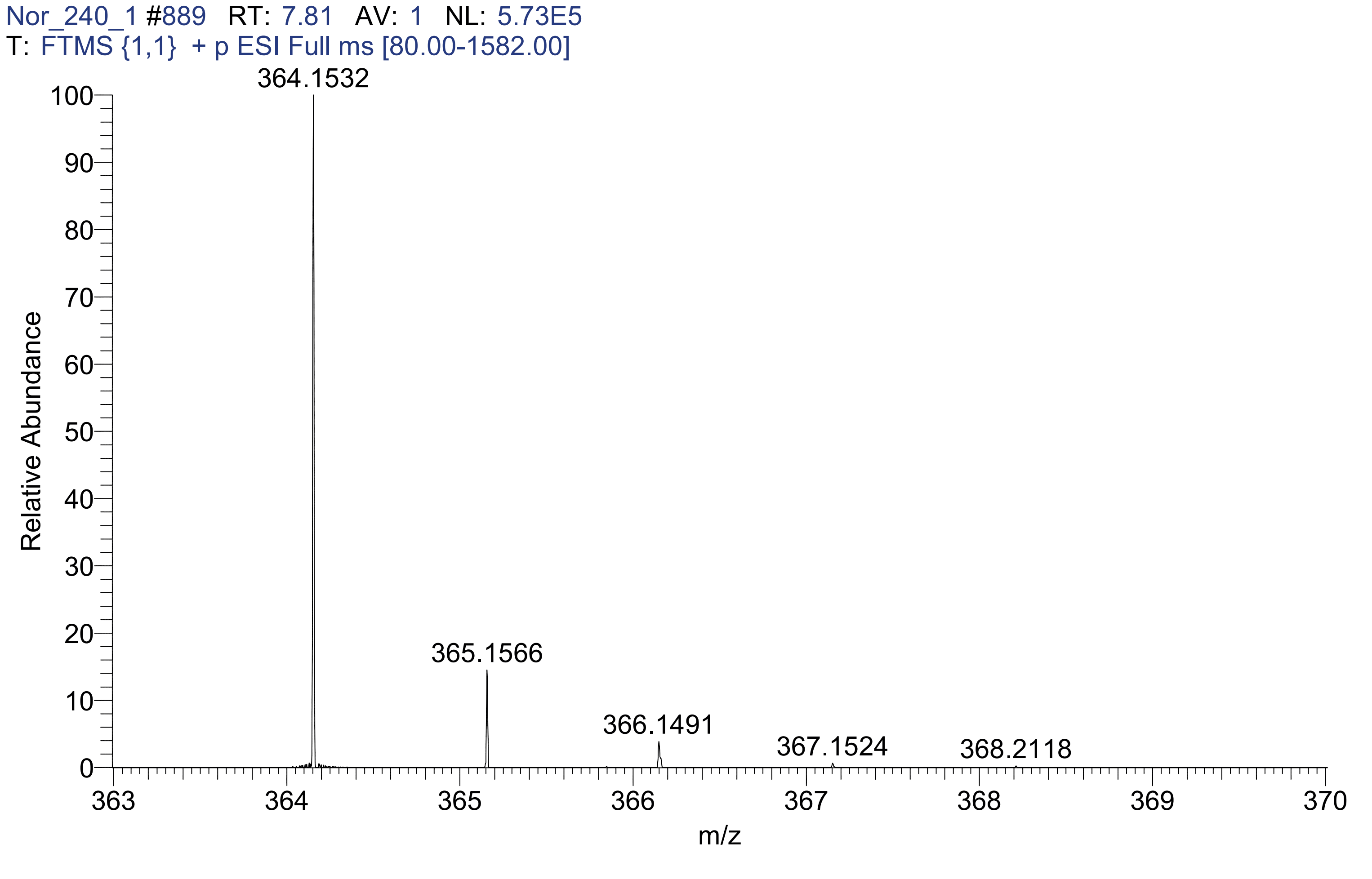

ACVS reaction - substrates: L-α-Aaa, L-Cys, L-Leucine
Expected m/z of AC-Leu tripeptide: 377.162 (M_i) + 1.008 (H^+) = 378.170

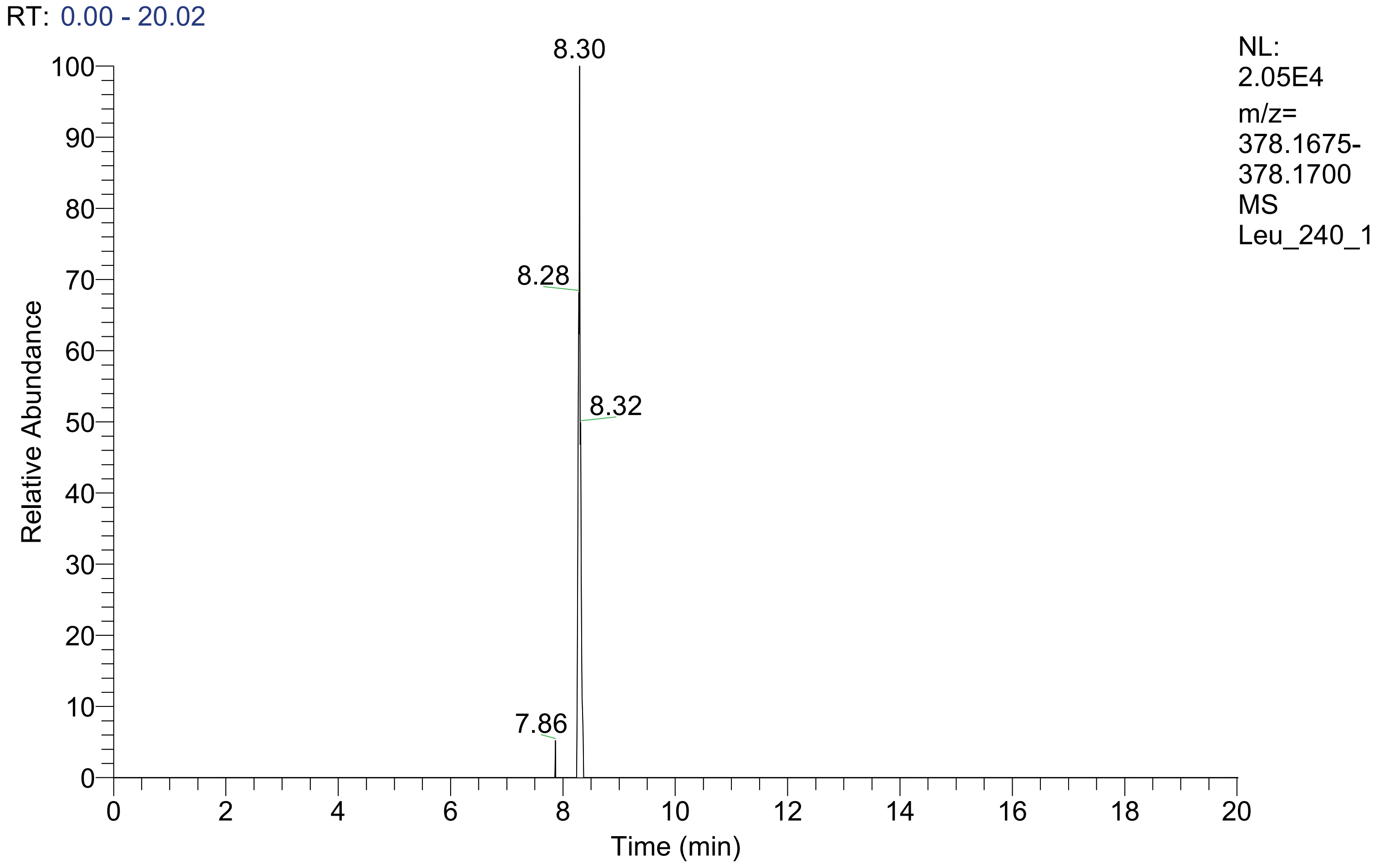

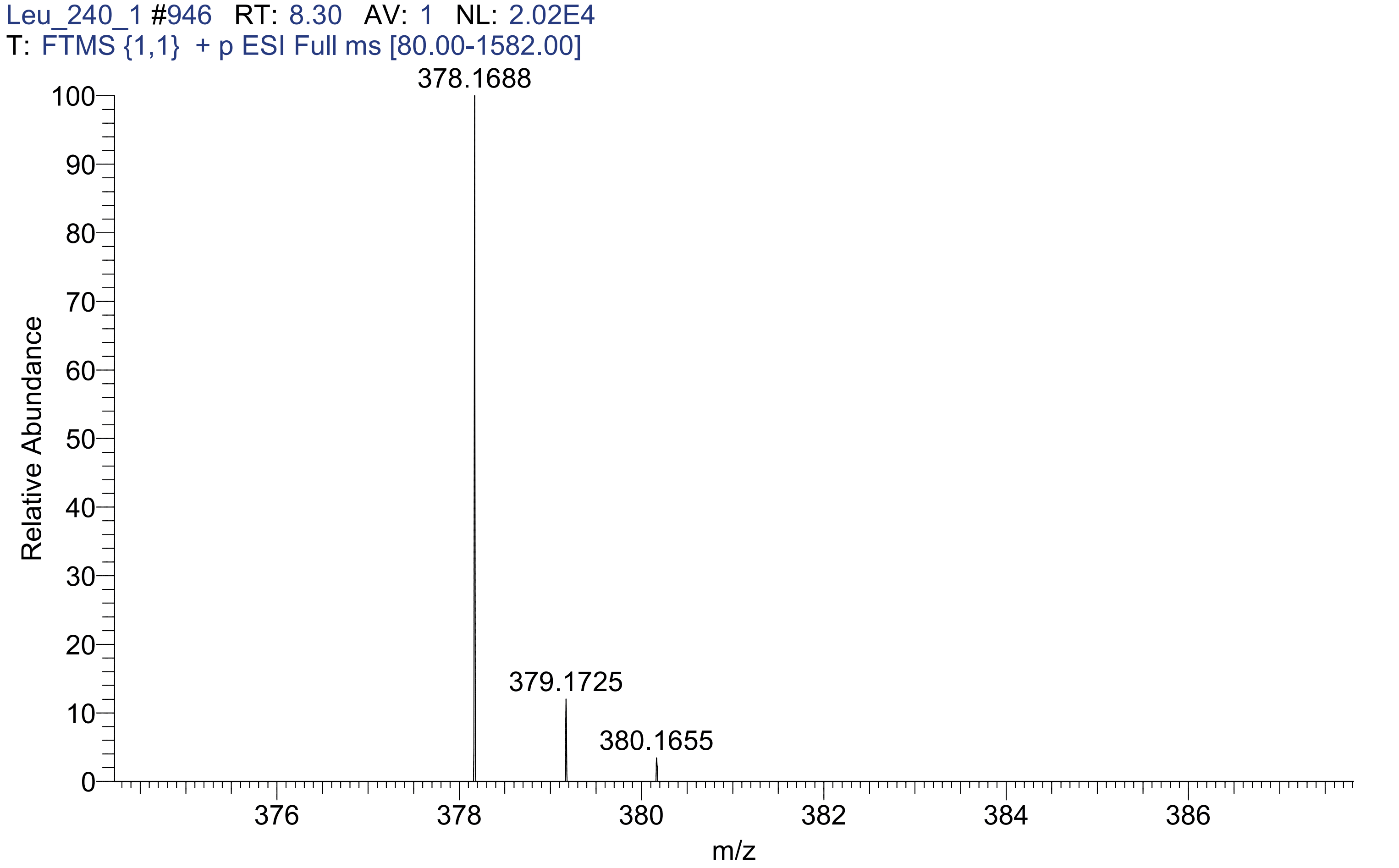

ACVS reaction - substrates: L-α-Aaa, L-Cys, L-Isoleucine
Expected m/z of AC-Ile tripeptide: 377.162 (M_i) + 1.008 (H^+) = 378.170

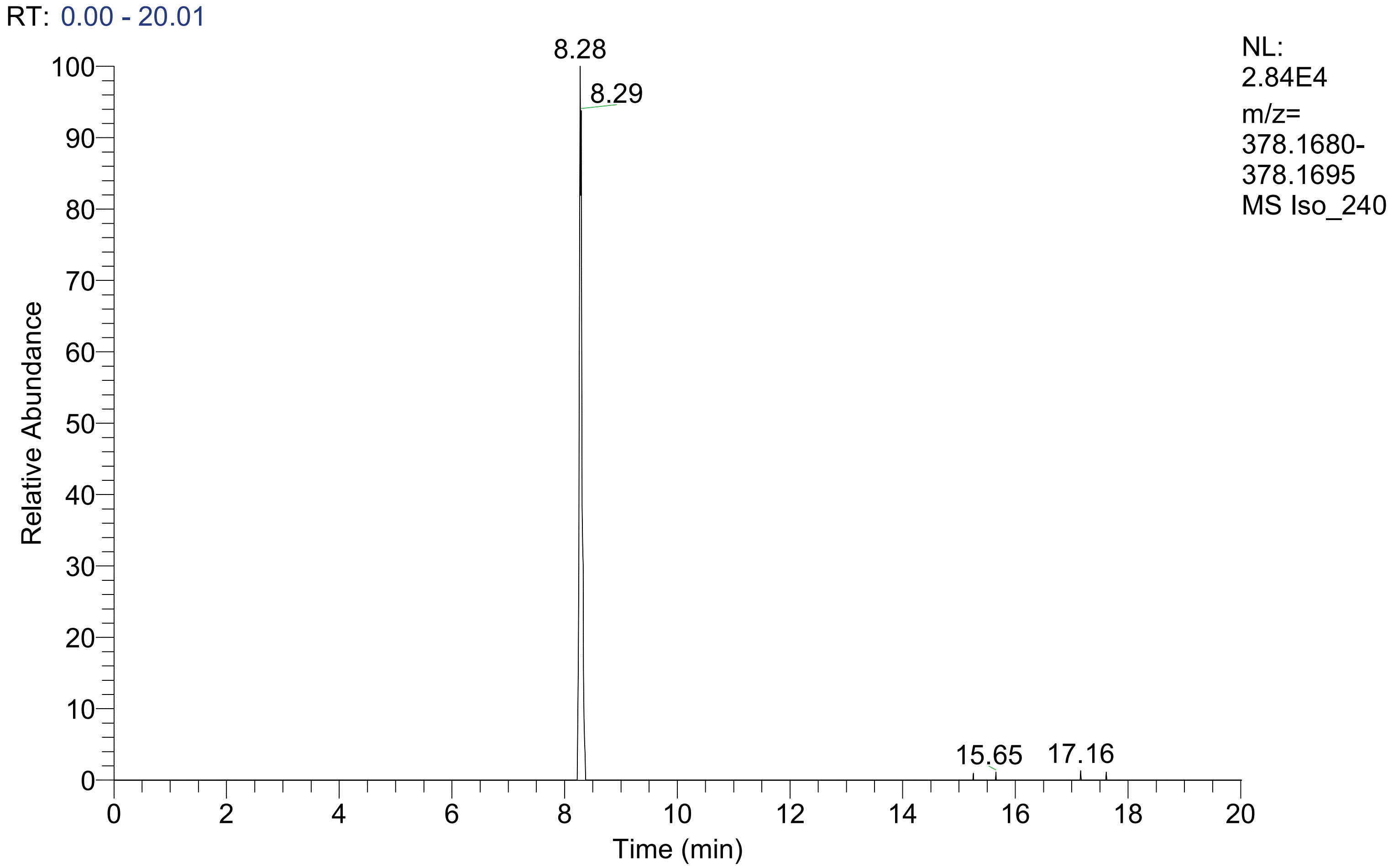

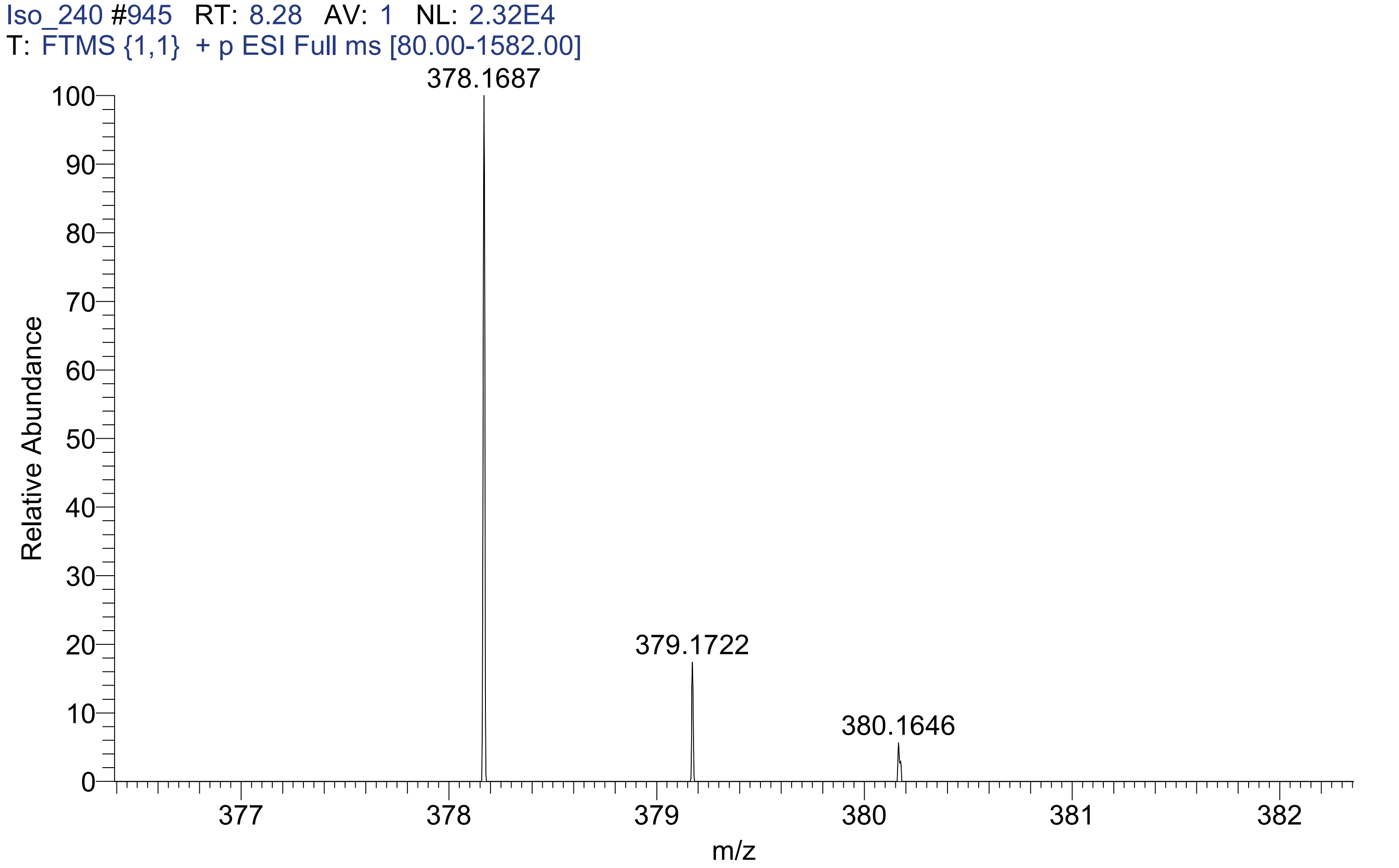

ACVS reaction - substrates: L-α-Aaa, L-Cys (no third substrate)
Expected m/z of AC-Cys* tripeptide: 367.087 (M_i) + 1.008 (H^+) = 368.095

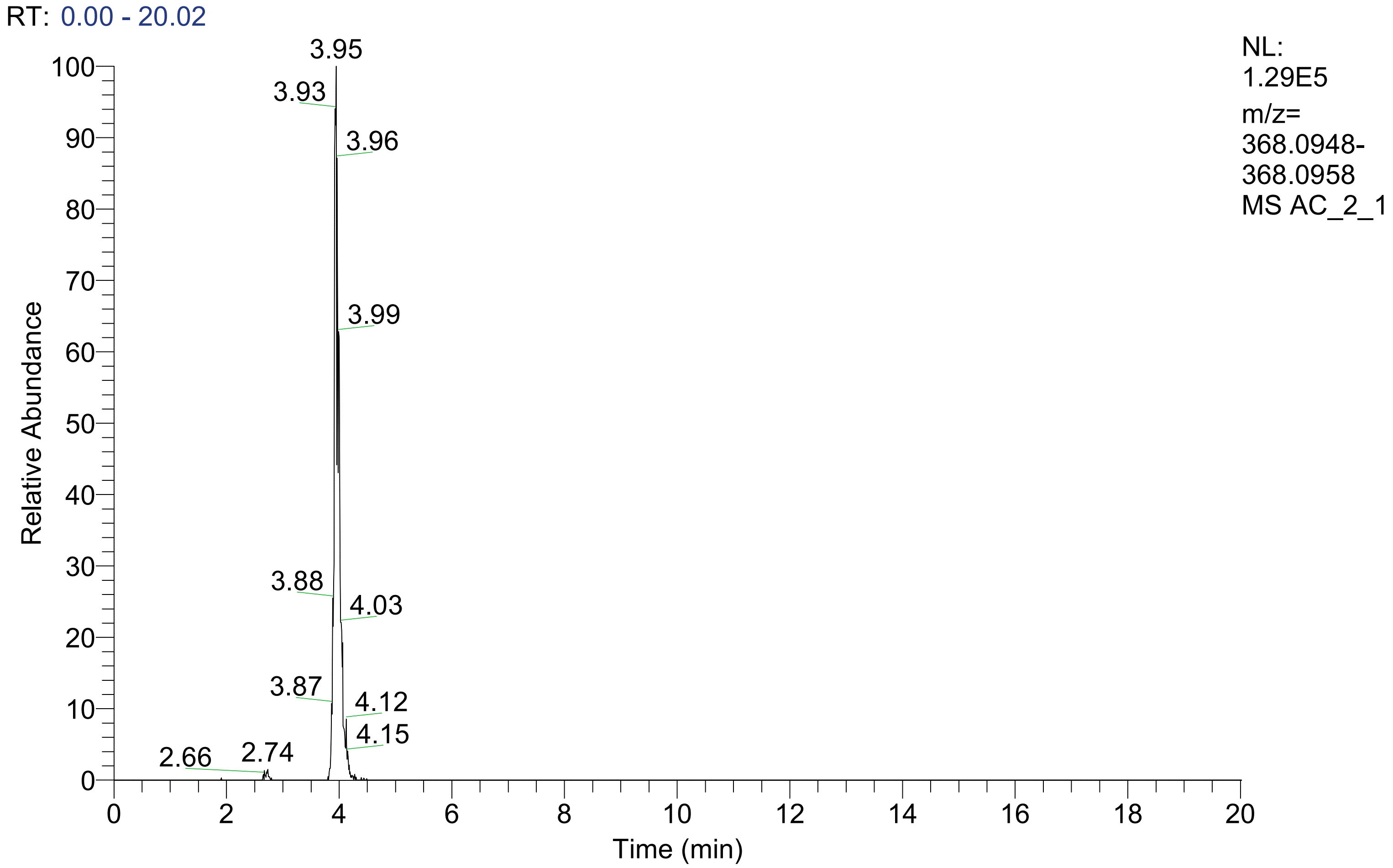

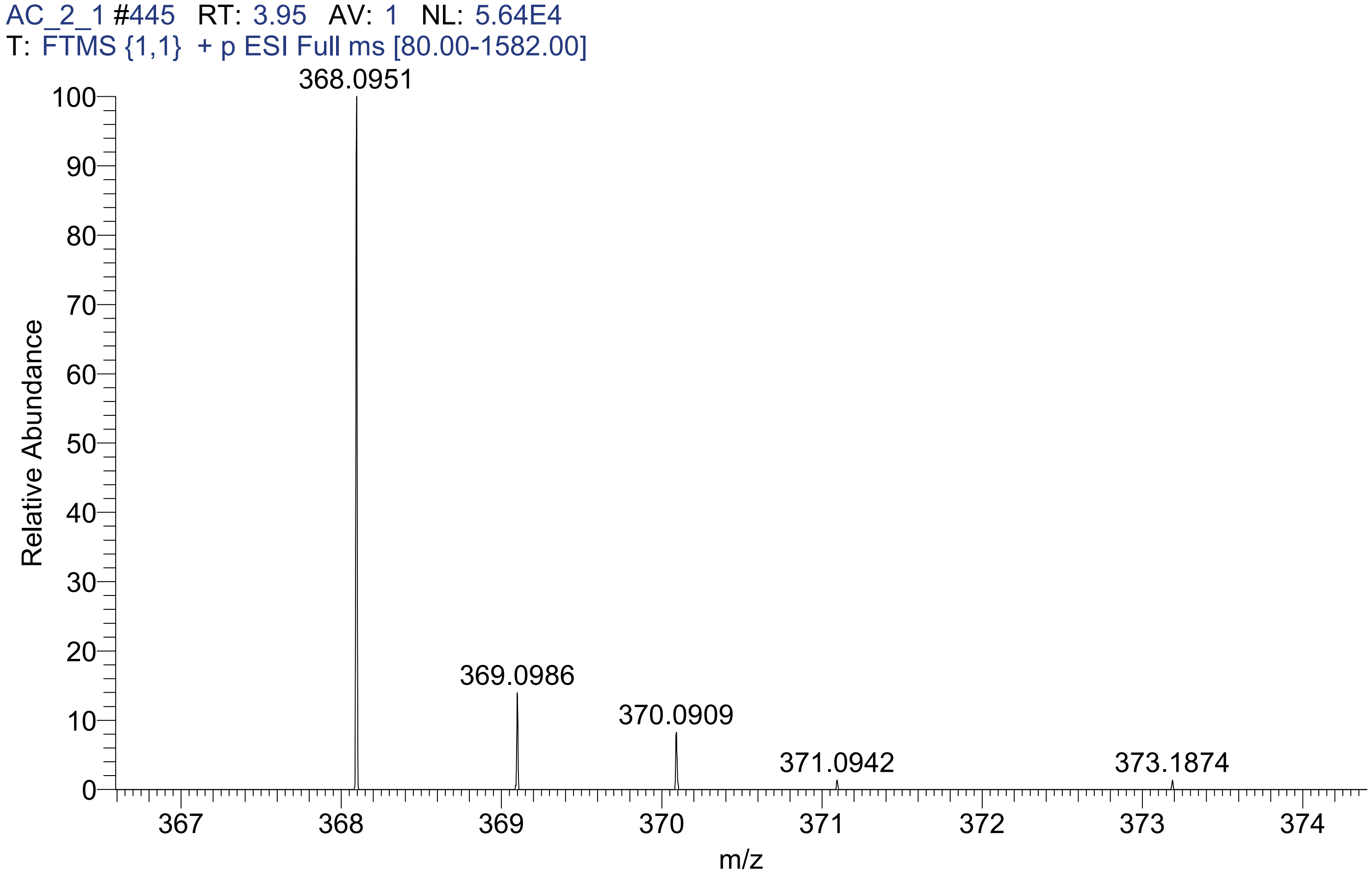

_PcANI_CVS reaction - substrates: - L-α-Aaa, L-Cys, L-Val
Expected m/z of ACV tripeptide: 363.146 (M_i) + 1.008 (H^+) = 364.154

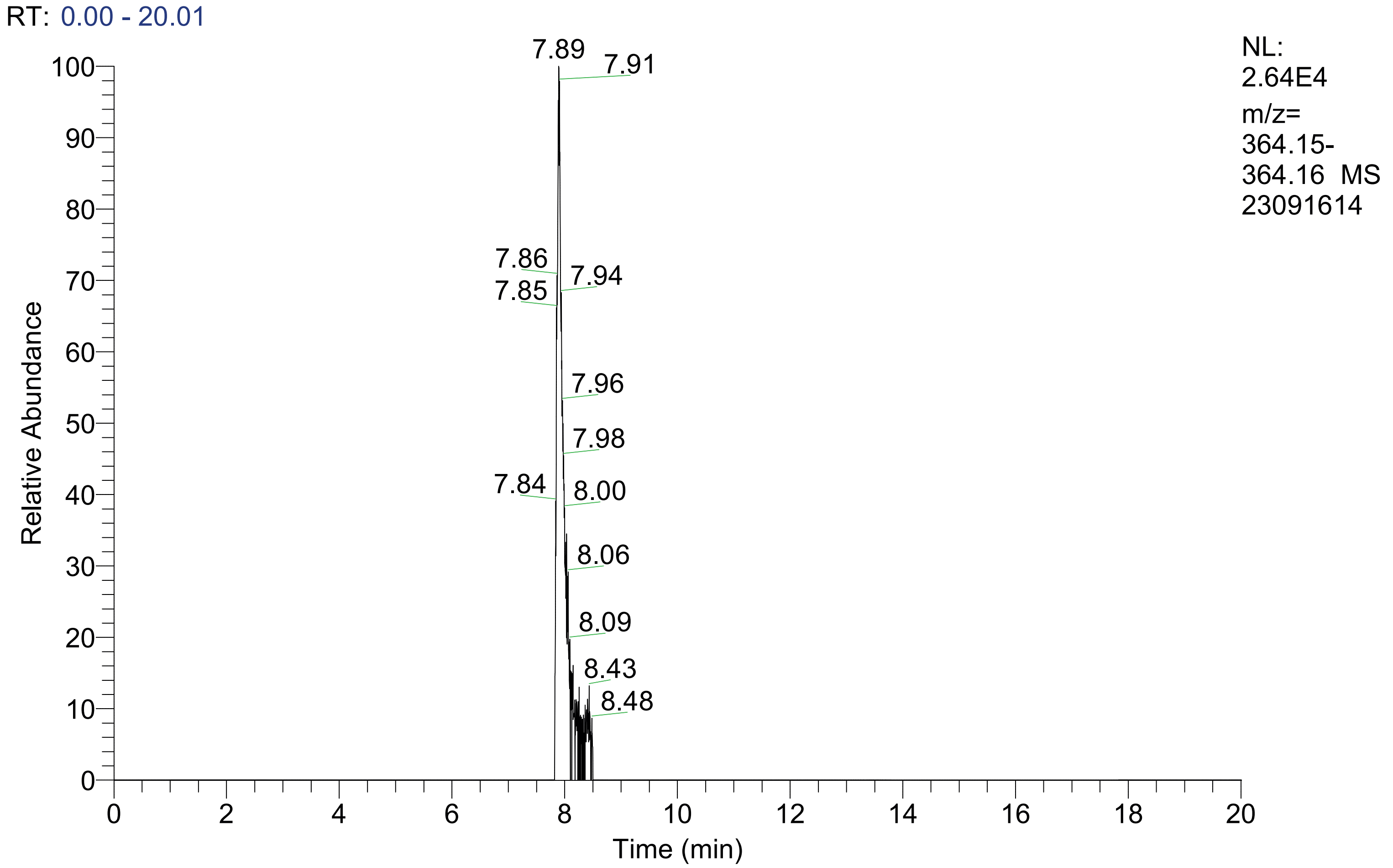

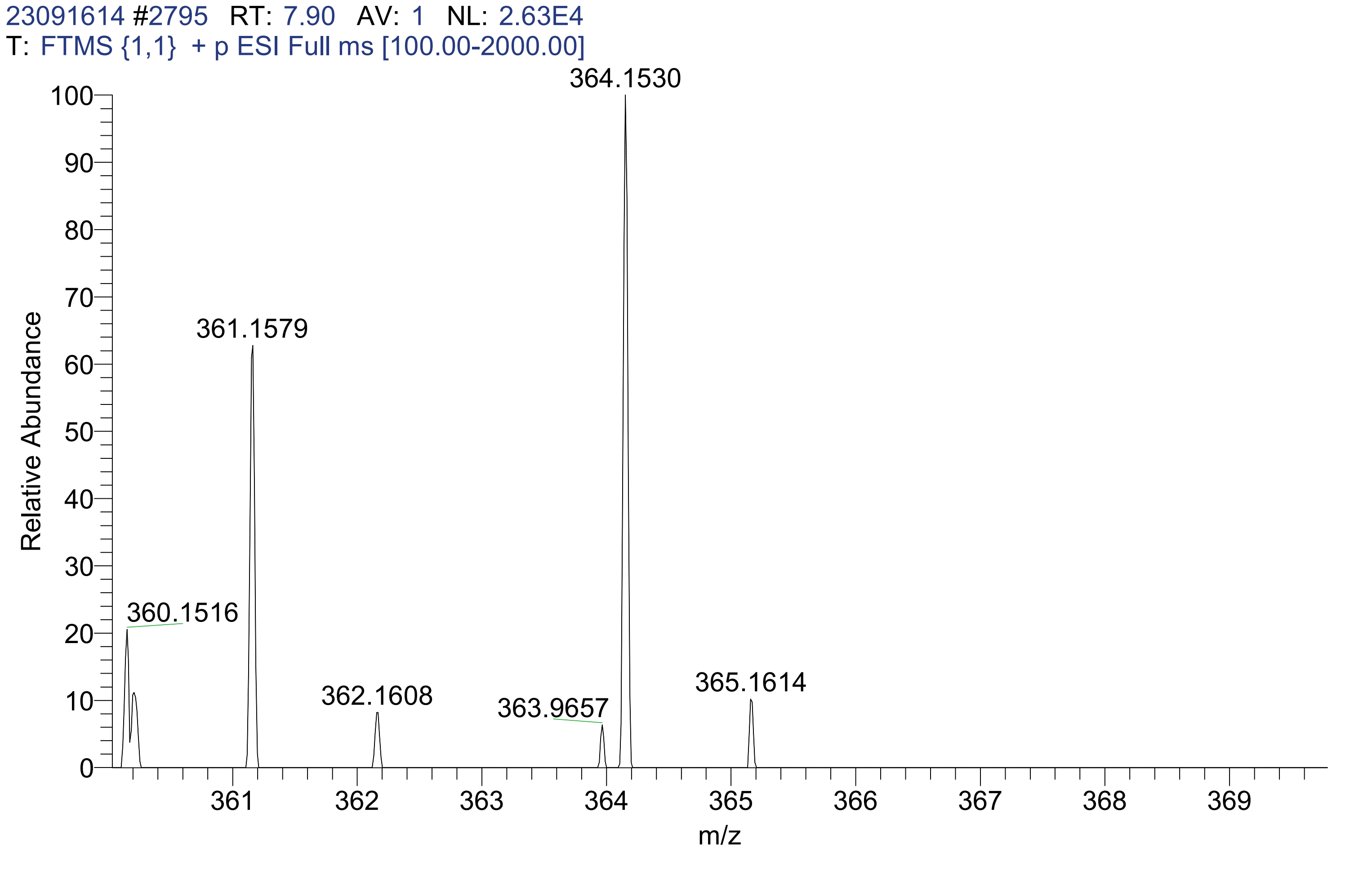

ACV synthetic standard
Expected m/z of ACV tripeptide: 363.146 (M_i) + 1.008 (H^+) = 364.154

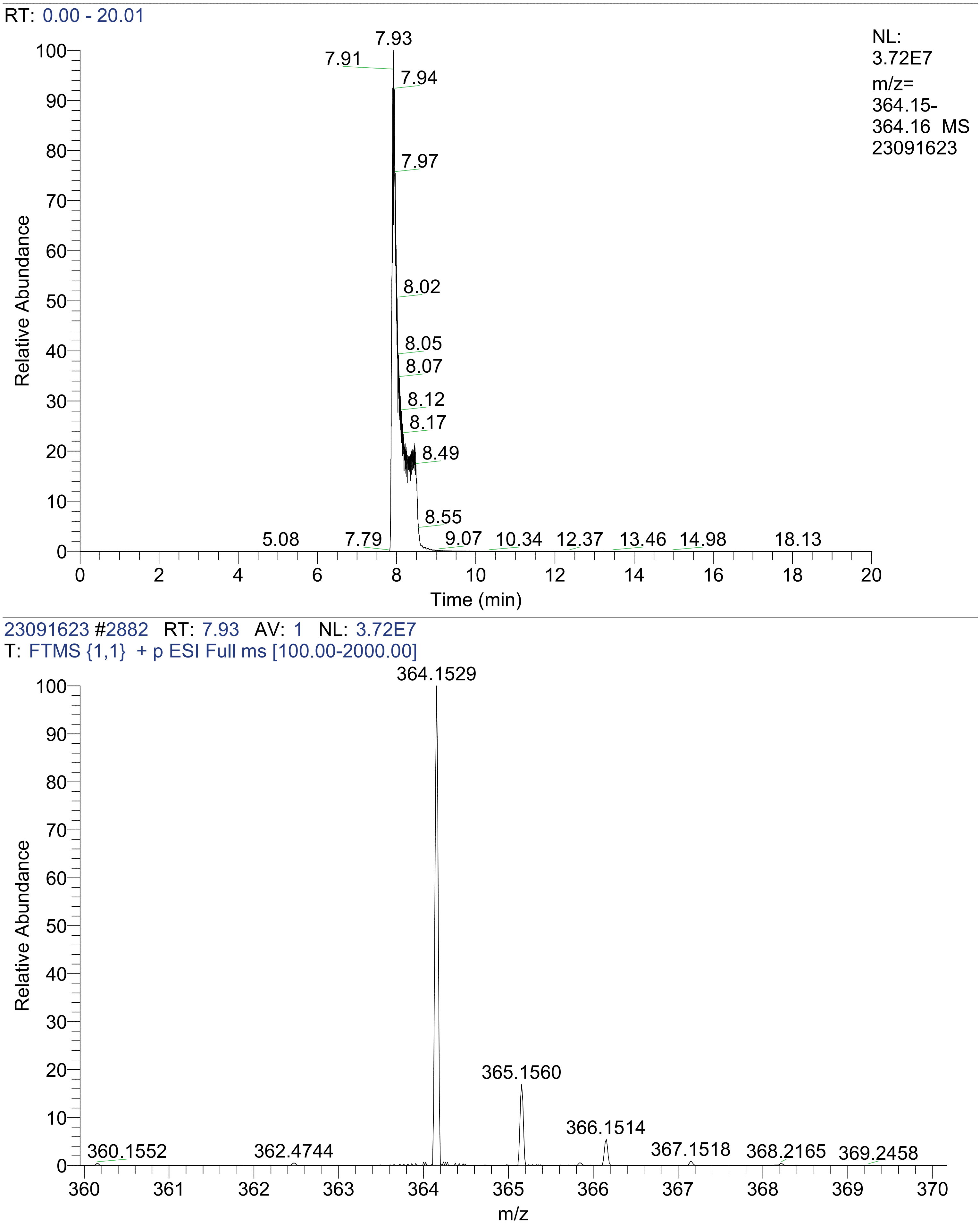

Supplement: S1 Appendix — The full chromatograms were filtered in accordance with the predicted m/z value of each tripeptide. The mass spectra of the resulting peaks were scanned for the presence of the expected compound. (PDF) [file pone.0231290.s004.pdf]
