# Supplementary material for: Biochemical characterization of the Nocardia lactamdurans ACV synthetase
Source: PLoS One. 2020 Apr 10;15(4):e0231290. doi: 10.1371/journal.pone.0231290 (PMC7147772; doi:10.1371/journal.pone.0231290)

Biochemical characterization of the *Nocardia lactamdurans ACV* synthetase

**Riccardo Iacovelli, Reto D. Zwahlen, Roel A. L. Bovenberg,
and Arnold J. M. Driessen**

S1_raw_images

SDS-PAGE images were taken with a Fuji ImageQuant LAS-4000 System and exported as high-resolution TIFF files using the AIDA image analysis software.

The following gel picture was used to generate Fig 2.

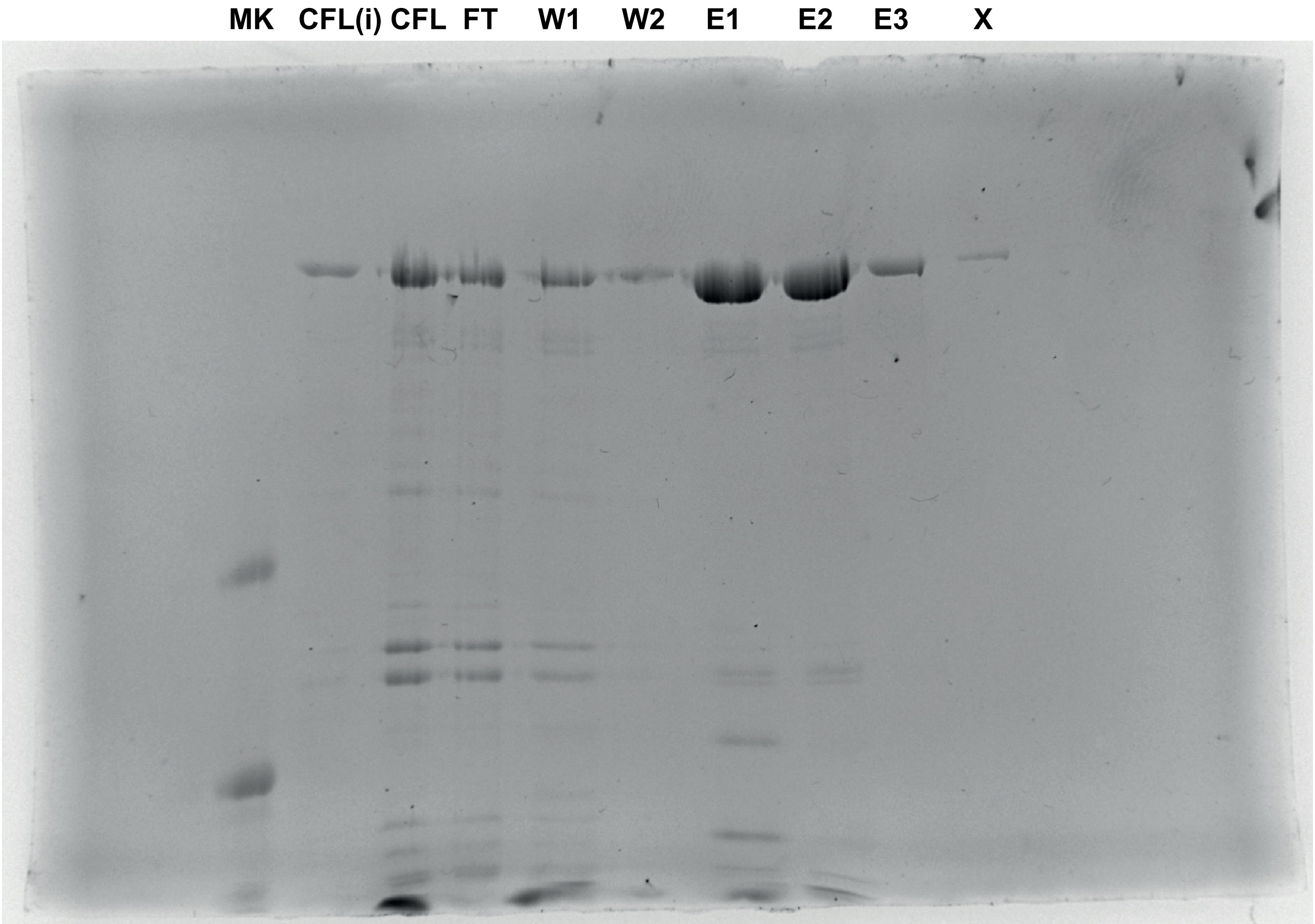

The following gel pictures were used to generate Fig 6A.

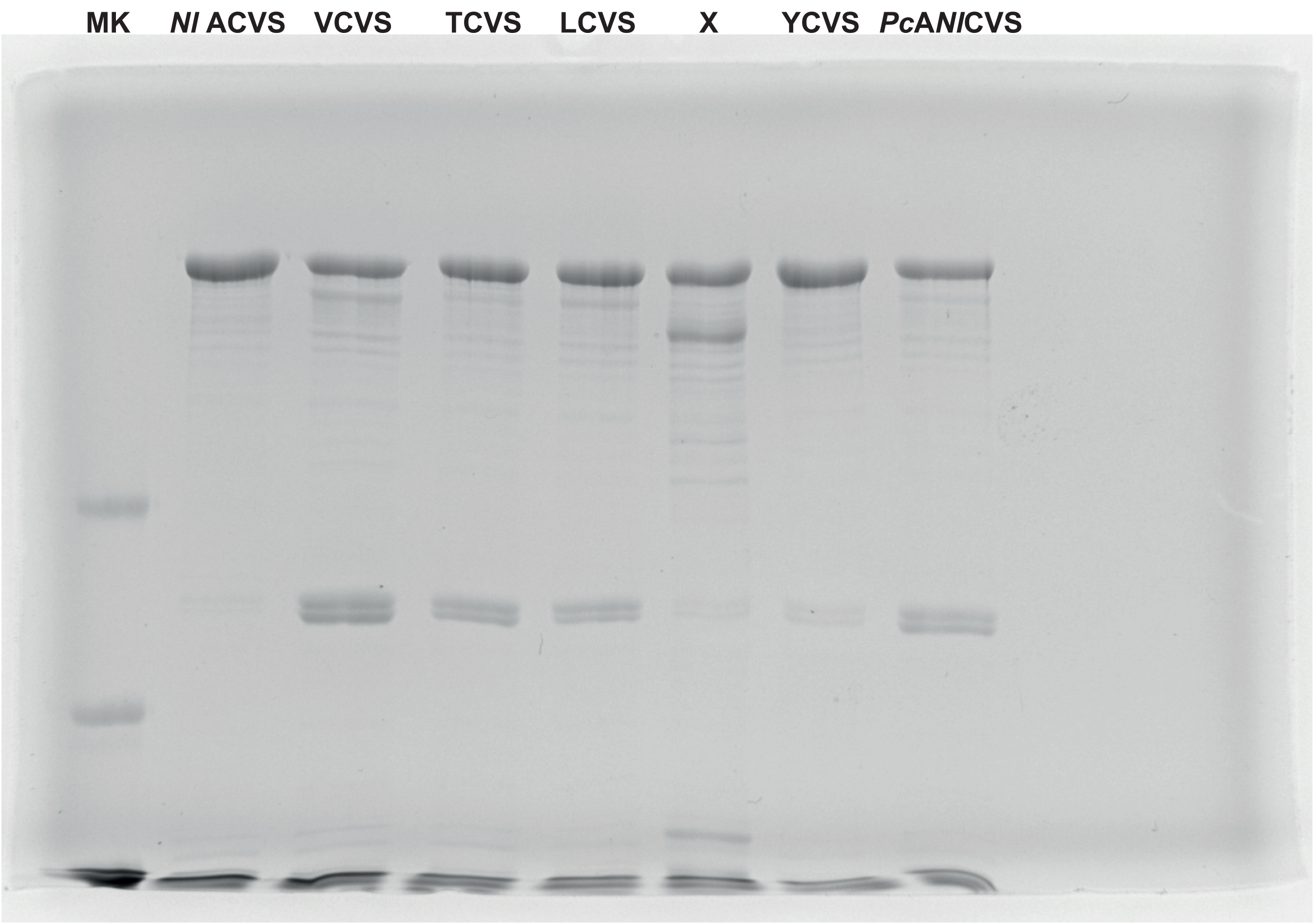

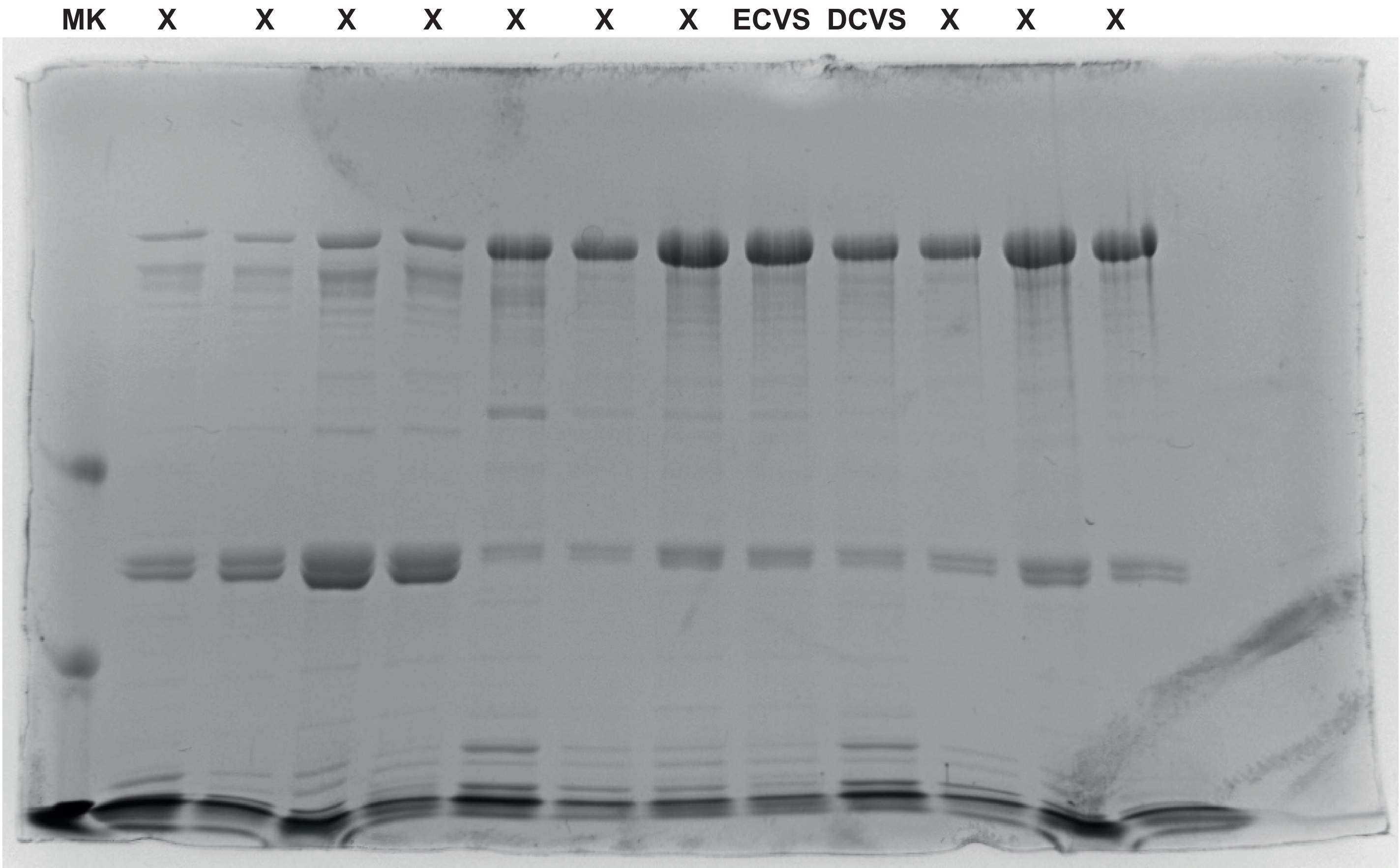

Supplement: S1 Raw Images — (PDF) [file pone.0231290.s005.pdf]
